# From dairy by-product to cell feed: milk whey for serum-free media and scaffold development in cell-based food production

**DOI:** 10.1038/s41538-026-00913-5

**Published:** 2026-06-03

**Authors:** Tamil Selvi Sundaram, Carlotta Giromini, Raffaella Rebucci, Federica Cheli

**Affiliations:** 1 Department of Veterinary and Animal Sciences (DIVAS), Università degli Studi di Milano, Via dell’Università 6, Lodi, Italy; 2 Coordinating Research Centre: Innovation for Well-Being and Environment (CRC I-WE), University of Milan, Milano, Italy; 3 Institute for Food, Nutrition and Health, University of Reading, Reading, United Kingdom

**Keywords:** Biochemistry, Biological techniques, Biotechnology, Microbiology

## Abstract

Cultivated meat is gaining attention as a sustainable source of animal protein, yet its research and development are constrained by limited access to serum-free media and scaffolding biomaterials. Milk whey, a nutrient-rich dairy by-product, exhibits biochemical and functional similarities to blood serum. This review examines the potential of whey as both a serum substitute and a scaffold material for muscle cell cultivation in cultivated meat and other cell-based production systems.

## Introduction

Muscle cell cultures established from either primary cells or immortalized cell lines, serve as valuable platforms for investigating cellular mechanisms underlying skeletal muscle phenotype^1,2^. As the primary cellular components of meat, adult muscle cells and their progenitors are increasingly explored as experimental models for developing in vitro meat cultivation strategies^2–4^. This emerging approach aims to produce animal meat under controlled laboratory conditions^5^, offering a sustainable alternative to conventional animal protein sources while addressing global challenges associated with rising demand for food, environmental impacts of intensive livestock production, and emergence of antibiotic resistance and zoonotic diseases^6–8^. The sustainability of cultivated meat production fundamentally depends on the use of ethically sourced cell culture components, including culture media, media supplements, and 3D scaffolds. The primary function of culture media is to support the proliferation and differentiation of muscle cells into mature myotubes. To achieve this, conventional media formulations are typically supplemented with fetal bovine serum (FBS) or horse serum (HS), which provides mitogenic factors required during different stages of muscle cell growth^9–11^. However, the use of animal sera is widely criticized in both preclinical research and cell-based manufacturing due to several limitations, including batch-to-batch variability and safety concerns associated with xenogeneic contaminants^12–14^. Although traditionally farmed meat is not entirely devoid of xenogeneic elements, the unethical methods employed in serum collection, its high cost, and the current regulatory framework governing FBS collection limit scalability and consumer acceptance, rendering such supplements unsuitable for cultivated meat applications^15–18^. Hence, plant^15–17^, algae^18,19^, insect^17^, microbial^20^ and food waste-derived^21,22^ extracts or protein hydrolysates are increasingly explored as FBS substitutes in formulating serum-free culture media for cultivated meat production^23^. While these alternatives can provide general nutritional support, they often lack essential components produced by animal cells for optimal muscle cell myogenesis, including key protein groups, growth factors, and hormones such as albumins, insulin, transferrin, fibroblast growth factor (FGF), insulin-like growth factors (IGF) and epidermal growth factor (EGF). Some plant extracts provide soluble proteins, including albumin and globulin fractions^24^, however, they may not function analogously to animal proteins. Consequently, these extracts are often combined with additional growth factors (e.g., FGF, IGF, EGF, insulin) and functional proteins (e.g., albumins, transferrin) to achieve outcomes comparable to conventional serum^23,25,26^. Microbial-derived substitutes may also require genetic engineering to produce missing bioactive components, introducing regulatory, safety, and public perception challenges^27,28^. Obtaining regulatory approval for food-grade use, particularly for novel plant or microbial derivatives not previously consumed at scale, adds further uncertainty and cost for industrial adoption^27^. In contrast, whey is a well-established dietary supplement that naturally contains many of these bioactive components^29–32^, making it a more effective and inherently supportive supplement for muscle cell culture. Previous studies have also demonstrated that whey proteins (WP) can support muscle cell proliferation and differentiation under serum-reduced or serum-free culture conditions^21,33–35^, highlighting their potential as a functional growth supplement. Milk and blood serum display several biochemical and functional similarities that are essential for supporting eukaryotic cell growth, development, and immune function^36–38^. During lactation, proteins, minerals and other biomolecules originating from either mammary tissue or bloodstream are selectively exchanged across the blood-milk barrier to support milk production. This contributes to a dynamic molecular resemblance between the two biofluids^37,39^. Similar to milk, whey is also enriched with macro- and micronutrients vital for cell growth^40,41^. Whey is the liquid by-product generated during the coagulation of milk in cheese production. Milk contains approximately 30–35 g/L of proteins, of which ~80% is casein and the remaining ~20% constitutes WP^40,42^. The dairy industry generates large quantities of whey annually, which is rich in organic matter. According to the 2022 European Union (EU) data on milk and milk products, approximately 55.9 million tonnes (Mt) of whey were produced within the EU alone^43^. Historically, whey was commonly discarded due to insufficient valorisation methods, contributing to significant environmental pollution and waste management challenges^44,45^. Over recent decades, advancements in membrane filtration and purification technologies have enabled the separation and enrichment of bioactive WP and peptides^29,46^. Consequently, whey has transitioned from an industrial waste into a high-value nutritional ingredient widely incorporated into infant formulas, sports beverages, and nutraceutical formulations^29,41,46^. Beyond its role as a growth supplement, WP such as β-lactoglobulin (β-LG), have also been explored as 3D scaffold materials for cell culture and tissue engineering applications^47,48^. β-LG can undergo gelation in aqueous solutions in response to stimuli such as changes in temperature and pH^47^. These properties support the use of whey components in serum-reduced or serum-free media formulations and the development of food-grade, edible scaffolds for cell culture applications. In this review, we outlined the general nutritional requirements for muscle cell cultivation, explored the biomolecular similarities between milk whey and FBS, and discussed their respective roles in muscle cell growth. Furthermore, we highlighted the potential of whey as an ethical, and sustainable, and multifunctional ingredient that can serve both as a serum substitute in cell culture media and as a scaffold material for sustainable cultivated meat productions.

## Myogenesis, muscle cell cultivation, and nutrient requirements

### Myogenesis

**Fig. 1:**
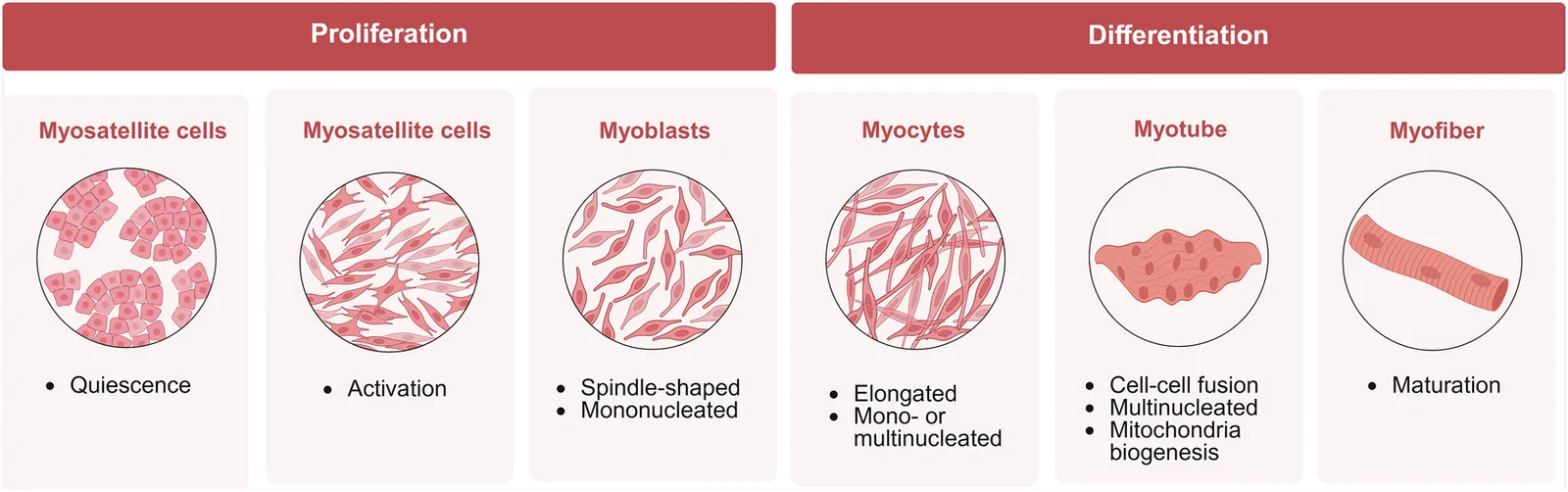
Different growth stages of muscle cell proliferation and differentiation. Figure created using BioRender.com.

Meat is primarily made up of skeletal muscles, which comprises approximately 90% muscle fibers and 10% connective and fat tissues^4^. Muscle fibers are formed through an intricate, multistep developmental process called myogenesis. During myogenesis, satellite cells or the myogenic precursor cells, which quiescently resides between the myofiber and the surrounding basal lamina are activated upon exposure to growth signals, injury, or self-renewal stimuli. The activated satellite cells proliferate into mononucleated myoblasts, which then differentiate and fuse to form longitudinal arrays of multinucleated myotubes. The myotubes mature into myofibers, forming the structural units of muscle tissues^49^ (Fig. 1). When establishing cultivated meat, it is important to consider that the composition and requirements of cell culture media vary during the different growth stages of muscle cells, such as proliferation, differentiation, and maintenance. In addition, culture media requirements vary depending on the starting cell type, and current research focuses on three categories of cells: pluripotent stem cells, unipotent stem cells, and somatic cells, each model with distinct nutritional needs, as reported earlier^50^. Much of the existing knowledge regarding media formulations that support in vitro myogenesis comes from studies performed on primary muscle cells and established muscle cell line models, such as the murine skeletal muscle C2C12 cell line^9,51,52^. These cells are cultured in absence of biochemical or mechanical signals from the surrounding tissues. Hence, when muscle cells are cultivated in a co-culture system with other cell type of meat such as adipocytes and fibroblasts or when they are cultivated in 3D scaffolds, the culture media composition may need to be tailored accordingly^53–55^. In general, cell seeding in culture vessels to the early growth phase, culture media is supplemented with mitogen-rich FBS to facilitate cell anchorage and proliferation^11,56^. When the cultures attain complete confluency at later growth phase, the concentration of FBS is either reduced or replaced with HS to establish a low-mitogenic environment for promoting cell-cell fusion and differentiation into myotubes^9,11,51^.

## General culture media requirements for muscle cell cultivation

### Basal media

Basal media are formulated to meet minimal nutritional needs, enabling prolonged cell survival and propagation in cultures^57^. One of the most widely used basal media is Dulbecco’s Modified Eagle Medium (DMEM), a modified form of Eagle’s Minimum Essential Medium (MEM), originally developed by Harry Eagle in the 1950s by optimizing the nutritional requirements of human cancer and mouse fibroblast cells^57–60^. Ham’s F10, F11, and F12 are also derived from Eagle’s medium^61^. Presently, DMEM has emerged a standard medium for cultivating diverse mammalians, including muscle cells. Its formula contains glucose, amino acids, vitamins, and inorganic salts, tailored to support specific cellular growth requirements^60^. The general DMEM requirements for myoblast propagation are outlined in the following sections.

### Glucose

Glucose is the primary source of energy for cellular growth and functions like proliferation, metabolism, and differentiation^62^. In glycolysis, a six-carbon glucose molecule is metabolized into two of each three-carbon pyruvate and ATP, which fuels most energy-requiring cellular process, including muscle contraction^62^. Glucose also provides carbon backbones for synthesis of macromolecule such as nucleotides, amino acids, and lipids^62^. Metabolically active adult muscle and muscle satellite cells grow optimally in DMEM supplemented with 1000–4500 g/L of glucose^63,64^.

### Amino acids

DMEM contains thirteen L-amino acids, including arginine, cysteine, glutamine, histidine, isoleucine, leucine, lysine, methionine, phenylalanine, threonine, tryptophan, tyrosine, and valine^58,59^. In particular, L-glutamine is provided in 10- to 100-fold excess (e.g., 2 mM for muscle cells) due to its pivotal role in cellular functions^57,65^. Skeletal muscle and brain are the primary sites of glutamine production. Owing to its large tissue mass, skeletal muscle produces the majority of circulating glutamine, which represents ~50-60% of the free amino acids within this tissue^66,67^. L-glutamine is readily converted to L-glutamate by mitochondrial glutaminase, serving as an amino group doner for biomolecule synthesis and ATP production via citric acid cycle^67^. L-glutamine also functions as a carbon source that cannot be substituted by glutamic acid or glucose^65^. Furthermore, it can serve as an alternative energy source in certain metabolically active cell types^66^ or under glucose-reduced culture conditions, as demonstrated in studies using human fibroblastic cells^68^. Besides protein synthesis, amino acids contribute to biomolecule synthesis, antioxidation, cell signaling, and act as sources of energy, carbon, and nitrogen^65^.

### Inorganic salts

DMEM contains seven salts, including Sodium chloride (NaCl), Potassium Chloride, Sodium Phosphate monobasic, Sodium Bicarbonate (NaHCO_3_), Calcium Chloride (CaCl₂), Ferric Nitrate, and either Magnesium Chloride or Magnesium Sulfate. NaCl and NaHCO_3_ are present in higher concentrations^57,59^ to mimic physiological conditions. In aqueous solutions, these salts dissociate into respective ions that functions as cofactors and mediators of enzymatic reactions, metabolism, cell signaling, cell adhesion, and muscle contraction^69,70^. Most cells grow well at a slightly alkaline pH (7.2–7.4) in a 5% CO_2_ incubator, buffered by NaHCO_3_^71^. Together with amino acids, inorganic ions regulate osmotic balance, cell volume, and transmembrane potential^69,71,72^.

### Vitamins

Vitamins are small organic molecules required in small amounts for intracellular process. They act as antioxidants^72^, enzyme co-factors, and prosthetic groups in metabolism^73^. Of the thirteen essential vitamins, DMEM includes six B-complex vitamins (thiamine, riboflavin, niacinamide, pantothenic acid, pyridoxine, folic acid) and two vitamin-like compounds (choline, inositol), indispensable for cell survival and growth^58,74^. B-complex vitamins regulate carbohydrate, amino acid, and lipid metabolism, and are involved in single-carbon transfer, and synthesis of heme, purine, and pyrimidines^73^. Vitamin-like compounds primarily support lipid transport and metabolism to maintain the structural integrity and signalling functions of phospholipid cell membrane^74^.

### Serum supplement for myoblast proliferation and differentiation

The use of serum supplements in cell culture media has evolved significantly since the mid-20th century. The earliest application of serum components for myoblast culture was reported by Albert Fischer in 1948, who demonstrated that basal media supplemented with 0.05% dialyzed chicken blood plasma could support the short-term propagation (4–14 days) of chicken embryo myoblasts^75^. Based on this work, in 1954 and 1955, Harry Eagle demonstrated the long-term propagation (4–8 weeks) of mouse myoblasts in media containing 0.5–2% dialyzed HS^59^, and additionally showed that fibroblasts and HeLa cells could be cultured in media containing 1% dialyzed HS and 5% dialyzed human serum^60^. A major advancement came in 1958, when Theodore T. Puck and colleagues demonstrated that supplementation of media with 15% FBS, together with careful regulation of pH and temperature, prevented mitotic inhibition in human skin fibroblast-like cells and Chinese hamster ovary epithelial cells^76^. This approach enabled cells to be maintained at high proliferation rates for 3–6 months without developing aneuploidy, establishing a reliable framework for sustained cell expansion while minimizing chromosomal abnormalities^76^. Since then, FBS has been a key component of cell culture system for over 60 years, supporting diverse research and pharmaceutical applications^13^. It offers a rich blend of essential nutrients for in vitro cellular attachment, expansion, maintenance, and long-term cryopreservation, and continues to serve as the standard supplement for most animal cell cultures to date^13,77,78^. Although alternatives such as newborn calf serum, calf serum, and donor bovine serum are available, FBS predominates due to its high fetuin content, which supports cell growth^79^, and its relatively low levels of immune components (e.g., Igs, complement factors), which minimize undesired immune responses that could otherwise compromise experimental integrity and the reliability of research or diagnostic outcomes^13,76,80^.

### Fetal bovine serum - collection, composition, and cellular functions

FBS is derived from blood plasma through a two-step process. Whole blood contains plasma along with cellular components such as erythrocytes, leukocytes, and thrombocytes. Plasma itself is composed of ~90% water, with the remaining fraction consisting of 6-8% proteins, carbohydrates, blood clotting factors, minerals, and hormones^81^. To derive FBS, blood is cooled and allowed to clot for separation into two distinct fractions, serum (FBS) and fibrin clot with blood cells. The serum fraction is collected by centrifugation for downstream applications^82^. FBS has a highly complex molecular composition, containing thousands of macro- and micronutrients, including proteins, amino acids, lipids, carbohydrates, growth factors (GFs), hormones, minerals, and other biomolecules originating from diverse cells and tissues (Table 1). Less than two decades ago, 79–91 proteins were identified in three commercial FBS lots using the nano LC-MS/MS analysis^83^. The total protein concentration of these FBS lots ranged between 0.032 and 0.042 g/L^83^. With recent advancements in LC-MS/MS analysis, about 200–400 proteins and thousands of small molecule metabolites were characterized^84^. Albumins (37.09%) and immunoglobulins (Igs) (35.42%) are the most abundant, followed by apolipoprotein A1 (8.31%), serotransferrin (3.49%), and hemopexin (1.54%), collectively accounting for 85.85% of bovine serum proteins^84^. Most serum proteins, except Igs, are synthesised and secreted by the liver. Serum albumins are versatile proteins, well recognized for their roles in transport, antioxidant defense, pH regulation, protection against lipid peroxidation, and the support of cell proliferation and survival^85^. Albumins can covalently or non-covalently bind various biomolecules and ligands, such as fatty acids, amino acids, peptides, hormones, fat-soluble vitamins, metal ions, steroids, and pharmaceutical drugs, facilitating their intra- and intercellular delivery. Its antioxidant activity is attributed to sulphur-containing amino acids such as cysteine and methionine, which scavenge free radicals and prevent ROS generation through metal chelation^85^. Globulins are the second most abundant serum proteins, categorized into α, β, and γ (Igs) based on their electrophoretic mobility. Like albumins, globulins regulate nutrient transport, enzymatic reactions, and immunity. Igs are distinct as they are secreted by immune cells in response to infections, playing a central role in immune defense^86,87^. GFs and hormones in FBS are vital for myogenesis as they regulate the family of muscle-specific genes and proteins^10^. Cell cultures lack these nutrients and therefore require supplements like FBS, which provides at least thirteen GFs and fifteen hormones (Table 1). These small molecules regulate cell growth, metabolism, repair, signalling, and immune functions^10^. Hormones are produced and release into circulation by endocrine glands, while GFs are produced locally by different cell types. Studies using muscle cell models showed three families of GFs and hormones such as IGFs, FGF, and transforming growth factor beta (TGF-β) to have major effects on myogenic differentiation^10^. They control differentiation by regulating expression of myogenic regulatory factor genes, particularly myogenin^10^. TGF-β and FGF are potent differentiation inhibitors, while IGF stands out for multifaceted effects^10,88,89^. Specifically, IGF subtypes, including IGF-I, IGF-II, and insulin, promote proliferation, differentiation, nutrient uptake (glucose, amino acids, nucleosides), and macromolecule synthesis (protein, nucleic acid)^10,79,89,90^. FBS also contains various extracellular matrix glycoproteins (collagen, fibronectin, fibrinogen, laminin, integrin) that contribute to cell structure organization, signalling, and adhesion (cell-cell/-matrix), supporting tissue morphogenesis, differentiation, and homogenesis^91^. Notably, integrin-laminin interactions regulate cell adhesion, migration, proliferation, differentiation, and phenotype stability^91,92^. Fibronectins are critical for cell migration, wound healing, and inflammatory responses, highlighting multifunctional roles of glycoproteins in cellular dynamics^91^.

**Table 1:** Comparison of the nutritional components of fetal bovine serum and milk whey

Nutrients	Fetal bovine serum	Milk whey
**Proteins**	Serum albumin; fibronectin; fibrinogen; hemoglobin β fetal chain; laminin; collagen; integrin β-1; prothrombin; inter-α-trypsin inhibitor; hemopexin; haptoglobin; serum spreading factor; hemoglobin α/β chain; apolipoproteins (e.g., Apo A-I, Apo A-II, Apo A-IV); and members of the globulin family (α, β, γ), including α-globulins (e.g., α1-antitrypsin, α2-macroglobulin; α2-antiplasmin; antithrombin-III, α2-HS-glycoprotein, α1-lipoprotein, vitamin D-binding protein, transcortin, kininogen), β-globulins (e.g., plasminogen, β2-glycoprotein I, transferrin/hemiferrin, serotransferrin, β1-lipoprotein), and γ-globulins (e.g., IgA, IgM, IgE, IgG, IgD, Fc receptors)^14,81,83,84,221,222^	Serum albumin; serum albumin precursor (Isoform 1); bovine serum albumin precursor; β-lactoglobulin; α-lactalbumin; lactoferrin; galactoperoxidase; glycomacropeptides; ferritin; lactadherin; fatty acid-binding protein; hemoglobin β chain; proteose-peptone; perilipin; α1-acid glycoprotein; fibrinogen α chain; SEA domain-containing protein; phosphorylase b kinase regulatory subunit; polymeric Ig receptor; Ig-like domain-containing protein; coactosin-like protein; SERPIN domain-containing protein; peptidoglycan-recognition protein; calreticulin; hemopexin; apolipoproteins (e.g., Apo A-I, Apo A-II, Apo A-IV, Apo B, Apo E, serum amyloid A); GM2 ganglioside activator; plastin-2; zinc-α2-glycoprotein; clusterin; leucine rich α-2-glycoprotein 1; and several casein protein fragments; histone proteins; complement proteins; uncharacterized proteins; and members of the globulin family (α, β, γ), including α-globulins (e.g., α1-antitrypsin, α1-antichymotrypsin, α1B-glycoprotein, α1-lipoprotein, α2-antiplasmin, α2-macroglobulin, α2-HS-glycoprotein, vitamin D-binding protein, thyroxine-binding globulin, antithrombin-III), β-globulins (e.g., plasminogen, β1-glycoprotein I, β2-microglobulin, serotransferrin, serotransferrin-like protein, transferrin variant), and γ-globulins (e.g., IgA, IgM, IgE, IgG)^29–31^
**Enzymes**	Alkaline phosphatase; alanine aminotransferase (ALT/GPT); aspartate aminotransferase (AST/GOT); cone cGMP-specific 3′,5′-cyclic phosphodiesterase α-subunit; lactate dehydrogenase; lactoperoxidase; phosphokinase; prothrombinase; γ-glutamyl transferase; and transaminases^14,83,221^	Alkaline phosphatase; lactoperoxidase; L-lactate dehydrogenase A/B chain; pyruvate kinase; glyceraldehyde-3-phosphate dehydrogenase; fatty acid synthase; biotinidase; xanthine dehydrogenase/oxidase; peptidase S1 domain-containing protein; protein disulfide-isomerase; α-1,4 glucan phosphorylase; and glutathione peroxidase^30,31^
**Carbohydrates**	Monosaccharides (e.g., glucose, fructose, galactose, mannose, ribose); glycolytic metabolites^14,221^	Monosaccharides (e.g., sialic acid, N-acetylhexosamine, fucose, arabinose, galactose, glucose, mannose, altrose, talose, myo-inositol, gulose); disaccharides (e.g., lactose, lactulose); oligosaccharides; polysaccharides (e.g., exopolysaccharides, amyloid)^29,223^
**Fatty acids and lipids**	Medium-chain fatty acids (e.g., lauric acid); long-chain fatty acids (e.g., arachidic, arachidonic, docosahexaenoic; eicosadienoic, eicosatrienoic, linoleic, α-linolenic, myristoleic, myristic, palmitic, palmitoleic, heptadecanoic, stearic, oleic, and vaccenic acids); triglycerides; phospholipids (e.g., phosphatidylethanolamine); cholesterol; ethanolamine; phosphatidylethanolamine^14,133,221,224^	Short-chain fatty acids (e.g., butyric acid); medium-chain fatty acids (e.g., caproic, caprylic, capric, lauric acids); and long-chain fatty acids (e.g., myristic, palmitic, stearic, linoleic, linolenic, oleic acids); phospholipids^29,225^
**Growth factors and cytokines**	Insulin-like growth factor (IGF) (e.g. IGF-I/-II); IGF-binding protein 2/4; prepro-IGF-I; transforming growth factor (TGF) (e.g. TGF-β1); endothelial cell growth factor (ECGF); epidermal growth factor (EGF); basic fibroblast growth factor (bFGF); fibroblast growth factor (FGF) (e.g., FGF1/2/4/6/9); glial growth factor; hepatocyte growth factor (HGF); interferons (e.g., IFN-β/-γ); interleukins (e.g., IL-1/-1α/-1β/-6/-15/-17); nerve growth factor (NGF); platelet-derived growth factor (PDGF)^10,14,81,83,221,224^	Insulin-like growth factor (IGF) (e.g., IGF-I/-II); IGF-binding protein 2; epidermal growth factor (EGF); betacellulin; **c**olony-stimulating factor (CSF); transforming growth factor (TGF) (e.g., TGF-α/-β/-β1/-β2); fibroblast growth factor (FGF) (e.g., FGF1/2), interleukin-1 receptor accessory protein^32^
**Hormones**	Insulin; adrenocorticotropic hormone; corticosteroids; follicle-stimulating hormone; glucagon; growth hormone; luteinizing hormone; thyroid hormones; parathyroid hormone; pituitary glandotropic factors; prostaglandins; prolactin; testosterone; thyroxine; vasopressin^14,81,221^	Growth hormone; estrogens; progesterone; corticosteroids; insulin; bombesin^226–228^
**Vitamins**	Vitamin A; vitamin C; vitamin E; and B-group vitamins (e.g., B1, B2, B3, B4, B5, B6, B6 precursor, B9)^14,221^	Vitamin A; vitamin E; β-carotene; and B-group vitamins (e.g., B1, B2, B3, B4, B5, B6, B9, B12)^29,208^
**Minerals**	P; K, Na, C1, Se; Ca; Mg; Fe; Zn; Cu; B; Sr; Li; As; Sb; Pb; Hg; Cd; Co; Cr; I; F; Mn; Mo; V; Ni; Sn^14,133,221,229^	K; Ca; Mg; Na; P; Se; Ba; B; Co; Cu; Cr; Fe; Li; Mn; Mo; Ni; Sr; V; Zn; Al^29,210,211^
**Amino acids**	Tryptophan; threonine; isoleucine; leucine; phenylalanine; β-alanine; tyrosine; threonine; methionine; lysine; cystine; valine; arginine; histidine; alanine; aspartic acid; glycine; proline; serine; glutamic acid; glutamine; citrulline; α-amino-n-butyric acid; cystathionine; taurine; hydroxyproline; 1-methylhistidine; 3-methylhistidine; ornithine^133^	Tryptophan; threonine; isoleucine; leucine; phenylalanine; tyrosine; threonine; methionine; lysine; cystine; valine; arginine; histidine; alanine; aspartic acid; glycine; proline; serine; gluten acid; glutamic acid; glutamine^230,231^
**Nonprotein nitrogen**	Urea; purines; pyrimidines; polyamines; creatinine; amino acids; ammonia^14,133,221^	Urea, uric acid; ammonia; amino acids; creatinine; creatine; phosphocreatine; choline; polyamines^147^

### Horse serum

HS is another commonly used cell growth supplement that provides essential nutrients and GFs necessary for cell growth. Unlike FBS, literature on HS is limited, particularly regarding its molecular profile, collection process, donor selection criteria (e.g., age, diet, sex, physiology), and suitability for research applications. Based on available information, HS is derived from healthy adult horse blood, mainly collected via venipuncture^93–95^. Standard blood collection guidelines should minimize trauma, bleeding, and hematoma formation^93,95^. HS is then separated from clotted blood using methods similar to FBS production. Unlike FBS, HS was reported to have high homogeneity between lots and to contain low levels of polyamine oxidase, the enzyme responsible for polyamine degradation. Consequently, polyamines are metabolized more slowly and accumulate, which has been associated with mitogenic effects^96^. HS was initially preferred over FBS due to lower cost and broader availability^97^, and moreover, it eliminates the need for animal sacrifice. It is commonly used for cultivating equine cells, but it also supports the growth of other mammalian cells^11,98,99^. HS is highly valued for its ability to stimulate myogenic differentiation of myoblasts or myogenic precursor cells into myotubes^11,98^. In 1960s, Yaffe and colleagues showed newborn rat skeletal muscle cells could optimally proliferate for months and differentiate into multinucleated fibers when cultured in media with HS or FBS^11,56^. Later study compared serum type (HS, FBS) and concentration on rat skeletal muscle cells (L8, L84)^11^. L8 cells cultured in basal medium with 10% HS exhibited a shorter proliferation phase and higher cell fusion within 1–2 days after reaching confluency than cells grown in medium with 10% FBS, which showed a longer proliferation phase and relatively lower fusion^11^. L8 cells showed poor growth and differentiation in 2% HS, but addition of insulin (1 × 10^−4^ g/L) significantly improved both. Furthermore, L84 cells exhibited a similar proliferation and differentiation profile regardless of the serum condition tested (10% FBS, 2% or 10% HS). This study further emphasized the significant impact of cell density on myogenesis. For example, switching from 10% FBS to 2% HS in L84 cultures seeded at higher density (1.2 × 10⁶ cells/plate) resulted in enhanced cell fusion than lower density (1.7 × 10⁵ cells/plate). These cells also showed increased creatine kinase (CK) activity and myosin expression, which are key myogenesis markers^11^. These findings laid the groundwork for optimizing and standardizing muscle cell culture conditions, particularly the widely adopted approach of stimulating myoblast proliferation with 10% FBS, followed by differentiation in 2% HS, as extensively validated using C2C12 myoblasts^9,51^. Another study demonstrated that 10% FBS led to a higher cell doubling rate in equine bronchial fibroblasts compared to HS, whereas HS more effectively promoted fibroblast differentiation into myofibroblasts^99^. These observations indicate that FBS enhances proliferation, likely due to its high mitogen content, whereas HS favours differentiation through high myogenic factor levels^10^. However, limited research highlights the need for further studies, ideally using high-throughput proteomics and metabolomics to achieve better understanding of serum effects on muscle cell behaviour.

### Limitations of FBS and HS use in cellular agriculture

Despite their widespread use, FBS and HS are largely criticized worldwide for applications in cell-based therapeutics and food production, due to ethical, safety, variability, environmental, economic, and regulatory concerns, as outlined below.

### Ethical concerns

FBS is obtained as a by-product of the meat industry from bovine fetal blood, collected via cardiac puncture in a closed system when a foetus is detected at slaughter^100^. EU regulations prohibit the transport and slaughter of cows during the final 10% of gestation^101,102^; however pregnant animals continue to reach abattoirs due to undetected pregnancies, illness, reduced productivity, or economic constraints, as reported by the European Food Safety Authority (EFSA) Panel on Animal Health and Welfare (AHAW) in 2017^101^. In parallel, there is growing concern regarding the potential suffering inflicted on foetuses during this process. According to the World Organization for Animal Health (OIE) and the American Veterinary Medical Association (AVMA), fetal death occurs from anoxia within 10-20 mins in utero post-slaughter, while the complete process from inspection to blood collection generally exceeds 40 mins^101^. Although fetal neurological development is incomplete and the capacity for consciousness or pain perception is limited^103^, the use of approximately 2 million foetuses annually to produce ~800,000 L of FBS, a number that continues to grow, raises further questions regarding the number of pregnant cows slaughtered, ethical practices, transparency, and the overall sustainability of FBS production^12,13,78^. Ethical issues also extend to HS procurement, despite it not being a slaughter by-product. For example, guidelines for equine blood collection are poorly defined^95^, and excessive withdrawal such as 25% of circulating blood volume, has been associated with adverse physiological effects, including tachypnea, distress, sweating, urination, defecation, and elevated heart and respiratory rates, with blood globulin levels taking up to 31 days to recover^104^. Standard practice therefore recommends limiting blood withdrawal to ≤10% of circulating volume, taking into account physiological factors such as pregnancy, obesity, and age^95^.

### Safety issues

FBS carries risks of contamination with mycoplasma, viruses, bacteria, endotoxins, and misfolded proteins like prions, posing significant hazards for cell culture applications, including the production of cultivated food, recombinant proteins, vaccines, and other therapeutics^77,105–107^. Noncytopathic viruses, prions, and mycoplasma are hard to detect than common contaminants like bacteria, fungi, or yeast^14,106^. Earlier, a variety of known and emerging bovine viruses, both single- and double-stranded genomes were detected in commercial FBS^107,108^. Molecular analyses detected mycoplasma and pestiviruses at frequencies of 14% and 84% in Argentinean irradiated FBS samples from 2015 to 2019^106^. According to another report, FBS contains 3.56 × 10^−7^ g/L of endotoxins in an average^109^. Contaminants can enter FBS during manufacture or via infected pregnant cows^106^. Although commercial FBS undergoes 0.1 µm triple filtration and γ-irradiation, complete removal of contaminants is not guaranteed, and non-irradiated products remain available^81,106,110^. Prions are causatives of progressive neurodegenerative diseases in humans and animals^111,112^. These diseases can be spontaneous, heritable, anthropogenic, or horizontally transmissible from animals to humans, such as through consumption of contaminated beef or beef products^111,112^. Apart from meat, blood serum from infected animals were regarded as a direct source of prions earlier, while milk poses no risk^112,113^. A notable example is the 1985 Bovine Spongiform Encephalopathy (BSE) outbreak in the UK and France, which highlighted human health risks associated with FBS use in vaccine production^105^. Strategies to prevent prion diseases, including pathogen surveillance, culling sick animals, banning high-risk materials, and excluding animals older than 30 months from the human and animal food supplies were proposed (https://www.cdc.gov/mad-cow/php/animal-health/index.html#cdc_generic_section_6-resources). Further, the EFSA adopted measures requiring EU member states to remove Specified Risk Materials, which may lead to prion diseases from food and feed chains as of October 1, 2000 (https://ec.europa.eu/commission/presscorner/detail/en/memo_05_263). With advancements in understanding of BSE, the OIE, EU, and United States Department of Agriculture (USDA) have indicated that BSE is not transmitted through bovine blood or blood products when proper slaughter practices are followed^114^. Despite these safety measures, prions detection in asymptomatic infected animals remains challenging^111^. On the other hand, HS has been utilized since 1894 in vaccine and antivenom production and in serotherapy for diseases such as tetanus, rabies, and diphtheria^115^. Although therapeutically valuable, equine serotherapy carries significant safety risks, including immune reactions ranging from mild allergies and serum sickness to potentially life-threatening anaphylaxis^115,116^. Analysis of five commercial HS batches from New Zealand, Brazil, and the United States using high-throughput sequencing identified viral families such as *Flaviviridae*, *Herpesviridae*, and *Parvoviridae*, with frequent detection of equine hepacivirus, equine pegivirus, and Theiler’s disease-associated virus^117^. Viral sequences were detected irrespective of the geographical origin of the serum batches^117^.

### Other important challenges

FBS production is inherently cost-intensive due to its complex collection process, stringent sterilization requirements, and high demand relative to limited supply. Its price has increased by 300% with growing demand in Asia and the Middle East^14,118^. As of June 2024, FBS prices ranged between 1400 and 1700 USD/L^119^. Even articles from the 1980s and 1990s reported rising costs of FBS and FCS, indicating a long-standing trend of escalating bovine serum prices^97,120^. As highlighted earlier, FBS availability depends on the slaughter rates of pregnant cows^101^ and harvest volumes are influenced by factors such as herd size, beef/dairy market trends, feed costs, government policies, and disease outbreaks^80,118,121^. Its composition further varies with donor diet, age, physiology, and exposure to hormones, antibiotics, or veterinary drugs, compromising reproducibility and translatability of experimental outcomes^14,76,80^. Additional variability arises from seasonal and geographical changes, even within batches from the same manufacturer, as each lot originates from different animals^105^. A recent study reported differences in metabolic profiles, immune responses, and cytokine secretion in lipopolysaccharides (LPS)-challenged epithelial cells (HCT-8 and HT-29) cultured in FBS from different suppliers^122^. Similarly, FBS concentration and composition can substantially alter cellular processes, including proliferation and differentiation^11,21^. FBS composition was shown to influence engineered tissue thickness, induce spontaneous artefacts that mimic cellular activity, alter cellular phenotype and responsiveness of cell surface receptors to specific compounds^78,109,123^. Although the production cost of HS is not clear, commercial pricing from major suppliers (e.g., Thermo Fisher Scientific, Sigma-Aldrich) suggests that HS is less expensive than FBS, likely due to its simpler collection process and broader availability. Further, HS exhibits considerable batch-to-batch variability, and its composition and concentration influence cellular functions^124,125^. For example, variability in HS lots was reported to impair self-renewal in multipotent murine hematopoietic progenitor cells, an effect linked to higher levels of phospholipase A2-derived fatty acid products in different batches^125^. Traditionally, researchers attempt to mitigate this variability by pretesting and stocking serum batches that yielded optimal results^109,125^, a time- and cost-intensive process. As serum is a blood-derived product, the European Medicines Agency (EMA), the Food and Drug Administration (FDA), and the USDA oversee regulatory frameworks under which bovine serum intended for use in the manufacture of biological and medicinal products must undergo quality-controlled production processes, consistent with Good Manufacturing Practice (GMP) or equivalent standards to ensure safety, quality, traceability, and sustainable sourcing^114,126–128^. Accordingly, all FBS batches must be tested and/or treated (e.g., heat inactivation or γ-irradiation) to ensure the absence of adventitious agents (e.g., prions, viruses, mycoplasma, bacteria, fungi) and exotic livestock diseases. Manufacturers must also provide batch-specific information, including catalogue and lot numbers, country of origin, final batch volume, manufacture date, and shelf life to enable complete traceability of serum origin^126^. Although these guidelines primarily focus on bovine serum, the EMA recommends applying the same standards to serum from other species (e.g., horse)^126^. Further, the EU Reference Laboratory for Alternatives to Animal Testing (EURL ECVAM) Scientific Advisory Committee and the Organisation for Economic Co-operation and Development (OECD) strongly recommends the use of serum-free, chemically-defined media formulated with non-animal alternatives to reduce variability associated with serum supplements in current and emerging in vitro methods^13,123,129^.

### Sustainable alternatives to animal serum in cell cultures

**Fig. 2:**
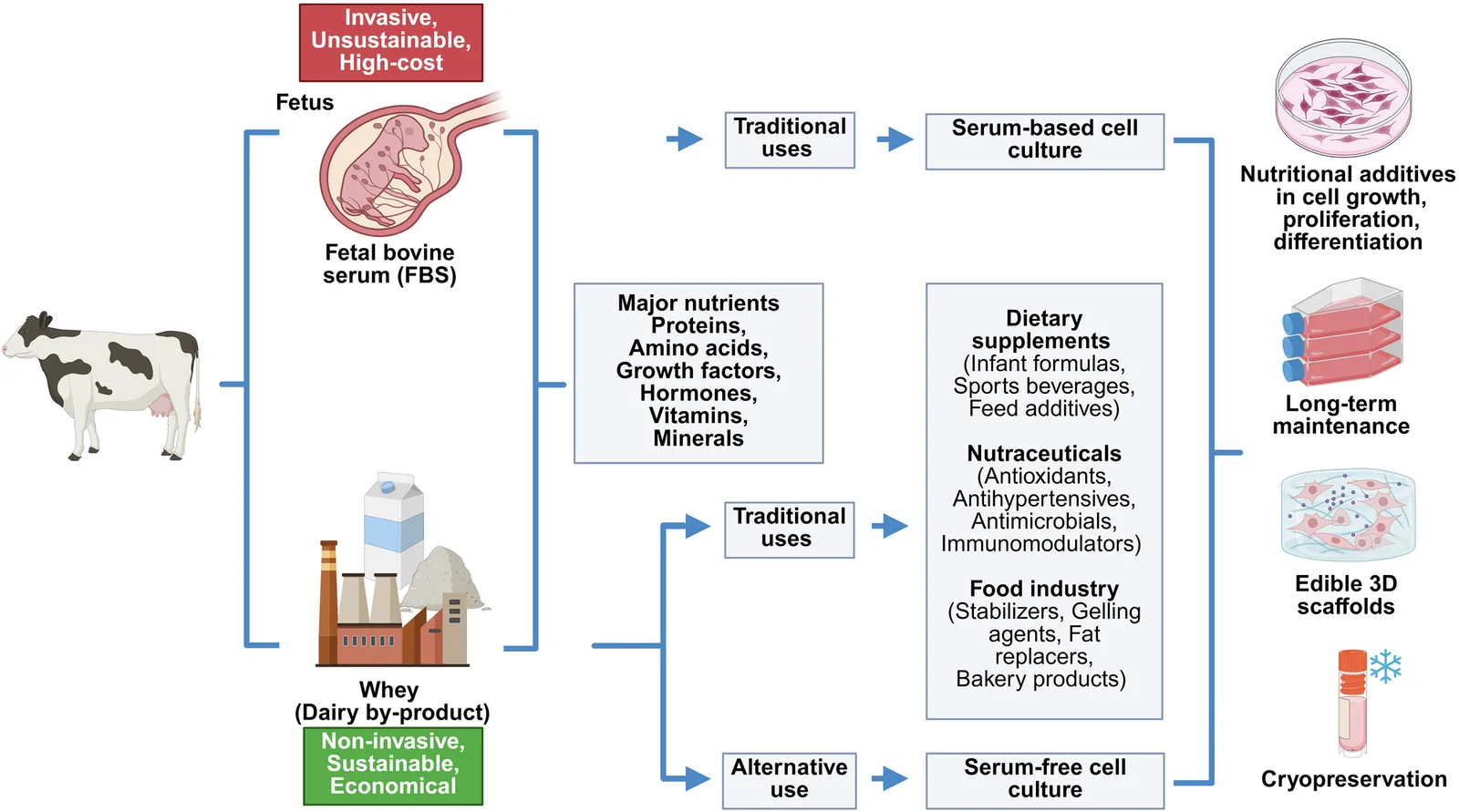
Overview of the traditional uses of whey by-products and their emerging potential as fetal bovine serum (FBS) alternatives for cell growth, maintenance, scaffolding, and cryopreservation in cultivated meat applications. Figure created using BioRender.com.

Cellular agriculture focuses on producing sustainable food products, including meat and milk, without unethically sourced animal components. For decades, limitations associated with the use of animal serum-based supplements in cell cultures have driven the ongoing search for safer, sustainable, and ethically acceptable alternatives^80,105,130^. While serum supplements pose challenges in laboratory-scale cultures, their impact is even more pronounced in industrial-scale cellular production due to their limited availability and high cost. For example, the first cultivated meat hamburger developed by Mark Post costed approximately €250,000 and relied on FBS-containing media, with culture media alone accounting for over 95% of the total production cost^131,132^. Similarly, another study estimated that FBS represents nearly 60% of cell culture media costs^133^. These limitation highlights the critical need for alternative, widely available, and cost-effective media supplements. Consequently, various animal-derived and animal-free hydrolysates were developed as potential substitutes for incorporation in serum-free media (SFM)^134^. Examples of animal-derived hydrolysates include human/bovine platelet lysates, hormones, bovine pituitary extracts, bovine vitreous humour, milk proteins, salmon, lymph, meat, and bacto- and peptic-peptone^14,21,119,130,134^. Bioactive substances such as silkworm sericin, earthworm extracts, and coelomic fluids have also gained attention for their regenerative, proliferative, and differentiation-promoting potential^14,135–137^. These components support the growth of different mammalian cells, including myoblasts, adipocytes, fibroblasts, epithelial cells, osteoblasts, lymphocytes, and mesenchymal stem cells^21^. To facilitate identification of FBS alternatives, the 3Rs Centre Utrecht developed an open-access FCS-Free Database (https://fcs-free.sites.uu.nl/), compiling peer-reviewed articles, product listings, and user experiences to FBS-free and chemically defined media. Upon searching keywords relevant to cell-based meat and milk (e.g., ‘Myoblasts’, ‘C2C12’, ‘Skeletal muscle cells’, ‘Adipocyte’, ‘Preadipocyte’, ‘Epithelial cells (Mammary)’, ‘MG (Mammary gland)’), the database listed many chemically-defined media and supplements, including B27, Multus Poliferum, AIM-V, Ultroser-G, Ult-G-AIM, and formulations containing bovine pituitary extract (e.g., PELOBiotech, PromoCell). While these chemically-defined formulations can enhance reproducibility of research outcomes and reduce the risk of serum-based contamination, data on their cost-effectiveness and ethical sourcing of animal components remains limited. On the other hand, animal-free hydrolysates are emerging as promising alternatives due to their efficiency, scalability, sustainability, and limited ethical concerns^25,134^. These include plant proteins^15–17^, insect proteins^17^, algal extracts^18,19^, microbial biomass and metabolites^20^, recombinant proteins, and nutrients extracted from food waste and industrial by-products^21,22^. Plant hydrolysates (e.g., soy, rice, chickpeas, flaxseeds, rapeseed, wheat) provide peptides, amino acids, and phytonutrients essential for cell growth^15–17^. Algal biomass, particularly from microalgae and cyanobacteria, provides fatty acids, antioxidants, and cell growth-promoting factors^18,19^. Microbial sources such as yeast extracts and bacterial hydrolysates offer scalable, consistent, and pathogen-free supplements that can reduce batch-to-batch variability^20^. Furthermore, food and agricultural by-products (e.g., vegetable peels, spent grain, cod backbone, eggshell membrane, chicken carcass) were shown to be nutrient-rich and effective for cell growth^21,22^, thereby supporting circular bioeconomy practices. Milk whey, a nutrient-rich dairy co-product, and its derivatives such as WP and peptides, can partially or completely replace FBS in muscle cell cultures (Tables 2 and 3)^21,33–35^. Owing to their high biological value and diverse bioactivities, including antioxidant, antimicrobial, and immunomodulatory functions, WP have been traditionally used in the food and feed industries, encompassing nutraceuticals, infant, sports, and animal nutrition, as outlined in Fig. 2^29,40,41^. In comparison to FBS ( ~ 1400–1700 USD/L)^119^, whey represents a low-cost dairy by-product, with current industrial market prices in Italy reported at approximately 1.75–20.50 €/1000 kg ( ~ 0.0021–0.0245 USD/L), with similar price ranges observed across other European countries^138^. Liquid whey effluent can undergo further processing steps such as pasteurization, thermal treatment, and membrane filtration to remove impurities or non-essential components (e.g., lactose) and to concentrate valuable proteins, peptides, and GF-rich fractions that support cell growth^139,140^. These additional processing steps may further increase production costs during large-scale implementation. For example, previously published data estimated that the manufacturing cost of α-lactalbumin (α-LA)-enriched whey protein concentrate (WPC; ~80% protein) is approximately 16.46–17.92 USD/kg^139^. Costs increase further when individual WPs are isolated owing to the operational costs associated with multistage purification processes. Compared with the overall cost of FBS, whey remains a significantly lower-cost, ethically favourable, and potentially sustainable alternative. Its rich molecular profile also highlights strong potential as a functional substitute to FBS in cellular agriculture applications.

**Table 2:** Role of milk- or whey-derivatives on muscle cell proliferation and differentiation

Study model	Compound assessed	Culture media concentration	Culture duration	Effect on muscle cells	Ref.
**Mouse C2C12 myoblasts**	Whey fraction from milk and colostrum	2.5–5 g/L	Day 0–30	↑proliferation (cell count, morphology) ↑differentiation protein (MYH4) and gene (Myh1, Myh2, Myh4, MyoG) expression ↑spheroid formation (spheroid size, DNA content) ↑mean cell doubling time	^212^
**Mouse C2C12 myoblasts and bovine primary myoblasts**	Milk and milk extract (100 or 1000 kDa)	10–25%	Day 3, 6, or 10	↑proliferation (cell and nuclei count, morphology) ↑ differentiation proteins (MyoG, MyHC)	^35^
**Mouse C2C12 myoblasts**	WP mixtures	WP mixture (1.25% β-LG, 1.25% α-LA, 1.25% BSA) or (0.07% β-LG, 0.15% α-LA, 0.15% BSA)	Day 1, 2, or 6	↑proliferation (mitochondrial activity) ↑cell proliferation, cell-cell fusion and elongation (microscopy) ↓cell membrane integrity (LDH release) ↑ differentiation enzymes (CK and CS activity)	^21^
**Mouse C2C12 myoblasts**	WP serum	5 g/L	1, 4, or 24 h	↑myotube protein synthesis (p-p70S6K (Ser389) and p-rpS6 (Ser235/236) proteins in rapamycin complex 1 (mTORC1) pathway) ↑myotube diameter (MyHC)	^213^
**Mouse C2C12 myoblasts, bovine and porcine myosatellite cells**	BSA with or without growth factors	0.025–0.4 g/L	Day 2, 3, or 5	↑proliferation (cell and nuclei count, microscopy)	^52^
**Bovine myosatellite cells**	Recombinant albumin	0.8–6.4 g/L	Day 3, 4, or 28	↑proliferation (cell and dsDNA count, cell doubling, microscopy)	^26^
**Mouse C2C12 myoblasts**	WPC 80.05%	0.1–0.4 g/L	24 h	↑differentiation enzymes (CK and CS activity) ↑differentiation genes (MyoD, Myf5, MyoG) and protein (MHC) ↑miRNA expression (miRNA-1, miRNA-133a, miRNA-206)	^33^
**Mouse C2C12 myoblasts**	Peptide derivatives of β-LG, α-LA, or BSA	2.5–5 mM	1 or 24 h	↑viability (cellular redox potential) ↓relative oxidative stress (free radical damage)	^34^
**Mouse C2C12 myoblasts**	LF with or without proliferation inhibitors	0.001–0.1 g/L	Day 0, 2, 4, or 6	↑viability (cellular redox potential) ↑proliferation proteins (low-density lipoprotein receptor-related protein (LRP)1, extracellular signal-regulated kinase (ERK)1/2)) ↑differentiation proteins (MyoG, MyoD, MyHC) ↑myotube hypertrophy (relative fusion index, myotube diameter)	^214^
**Mouse C2C12 myoblasts**	Whey	0–0.78 g/L	24 h	↑proliferation (mitochondrial activity) ↓relative oxidative stress (free radical damage)	^232^

**Table 3:** Impact of whey derivatives on edible 3D scaffold design and muscle cell growth for cultivated meat development

Model	Compound assessed	Scaffolding concentration	Culture duration	Effect on muscle cells	Ref.
**Mouse C2C12 myoblasts and porcine muscle stem cells**	WPI crosslinked with varying concentrations of transglutaminase and CaCl_2_	10% WPI, 0–160% transglutaminas, 0–5% CaCl_2_	Day 1, 3, 5, 7, or 9	↑cell adhesion, proliferation (cell count, morphology) ↑differentiation proteins (f-actin cytoskeleton, MyHC) ↑positive scaffolding properties (Young’s modulus, wettability, hydration, zeta potential, dense gel network)	^217^
**Primary Bovine Muscle Cells**	WPI or β-LG with or without varying concentrations of alginate and crosslinked with CaCl_2_	10% WPI or β-LG, 1.5–3% alginate, 1.0 M CaCl_2_	Day 1 or 3	↑cell adhesion, proliferation (mitochondrial activity, cell count, morphology) ↑positive physical-mechanical scaffold properties (homogenous crosslinking, stiff and denser gel networks)	^48^
**Immortalized bovine satellite cells**	WPI with or without alginate or glycerol and crosslinked with CaCl_2_	20% WPI, 5% alginate, 0.2% glycerol, 1 M CaCl_2_	Day 1, 4, or 7	↑cell adhesion, proliferation (mitochondrial activity, live cell count, morphology) ↑positive physical-mechanical scaffold properties (swell ratio, hydrolytic weight loss, stiffness and strength)	^218^
**Mouse C2C12 myoblasts**	WPI photo-crosslinked with alginate	10% WPI, 3% alginate	24 h	↑proliferation (mitochondrial activity, nuclei count) ↑protein (actin cytoskeletal protein, total cellular protein) ↑positive physical-mechanical scaffold properties (swell ratio, hydrolytic weight loss, stiffness and strength)	^220^

## Milk whey as an alternative cell growth supplement for cultivated meat

### Milk whey

Whey is a yellow-green liquid residue, generated after coagulation of casein from milk during cheese and yogurt making. It is rich in organic matter, loaded with lactose, proteins, GFs, and other vital elements (Table 1). Whey was discarded as industrial waste due to the lack of proper recycling techniques, emerging as a potential environmental pollutant until the mid-20th century. Concerns over its disposal were mainly linked to its high organic load, with a biochemical oxygen demand (BOD) exceeding 35,000 ppm and a chemical oxygen demand (COD) over 60,000 ppm^45^, which can rapidly deplete oxygen in soil and water bodies upon untreated disposal^141,142^. Technological advancements over the years enabled the concentration, fractionation, and purification of whey solids and whey-based ingredients. Especially, introduction of chromatography, membrane filtration, and electrodialysis, revolutionized whey processing and valorisation^142–144^. These methods control the physiochemical properties of whey by applying different processing techniques, such as coagulation with reagents, liquid-liquid extraction, dialysis, membrane filtration, isoelectric or thermal precipitation, to recover specific nutritional and bioactive constituents. According to the FAO, global milk production has increased over 77%, rising from 524 Mt in 1992 to 930 Mt annually since 2022. Within the EU, 160 Mt were produced as report by the EU Commission, and about 70% of the milk available to dairies is used for cheese and butter manufacture. The continuous growth of dairy industry has significantly increased the volume of whey effluent generated. Notably, 80–90% of milk used in cheese production is released as whey, which was estimated as 187–206 Mt globally each year, and projected to reach 203–241 Mt by 2030^44^. Consequently, whey processing has sustainably transformed this formerly troublesome by-product into an economically-valuable commodity, enhancing the profitability of dairy industries.

### Composition and types - Sweet and sour whey

Bovine milk accounts for ~81% of global milk production^145^. It consists mainly of water ( ~ 87%), lactose ( ~ 4.8%), fat ( ~ 3.7%), and protein ( ~ 3.5%), with traces of minerals, GFs, hormones, and vitamins^146^. Both milk and whey composition vary by species (bovine, ovine, or caprine) and their lactation stage (early, mature, or late)^40^. Fresh whey effluent from cheese-making contains 93.5–94.2% water, representing 80–95% of total milk volume^40,147^. The remaining 5.8–6.5% dry matter accounts for 50–55% of total milk solids^141,147^ and retains approximately 96% of lactose, 25% of proteins, and 8% of fat from milk^148^. Apart from animal species, the method of cheese production also influences whey composition. Cheese-making methods yield two types of whey, sweet and sour^141^. Sweet whey results from coagulation of milk with rennet (pH ~6.5), while sour whey results from coagulation with mineral or organic acid like acetic acid (pH ~4.6–5.2)^41,149^. Both processes hydrolyse casein, separating milk into solid curd and liquid whey^41,149^. Processing differences give sweet and sour whey distinct nutritional profiles and applications. Whey dry matter contains 66-77% lactose (depending on whey acidity), 8-15% proteins, 7–15% minerals, 4% fats, 3% lactic acid, and other minor components like amino acids, vitamins, and trace elements^141,149,150^. Sweet whey is largely used in dietary supplements and nutraceuticals owing to its high nutrient content, including proteins, amino acids, and B vitamins^147,151^. Conversely, sour whey is mainly utilized in animal feed and fertilizers due to its high lactose content, acidity, and unpleasant taste^151,152^.

### Commercial whey derivatives – concentrates, isolates, hydrolysates

Whey proteins (WP) are commercially available as whey protein concentrates (WPC), isolates (WPI), and hydrolysates (WPH), and are widely used as ingredients in protein-enriched food and nutraceuticals^40^. They are produced by concentrating proteins from pasteurized whey effluent using membrane filtration (MF) techniques such as microfiltration or ultrafiltration^144^. Membrane filtration is a low-temperature separation process in which porous membranes of varying pore size remove non-protein constituents, including water, lactose, fat, and minerals from whey^46,144^. Protein concentration of the retentate can be further increased by coupling with diafiltration, which yields WPC with 34–89% protein^40^. Higher protein purity is obtained through ion-exchange chromatography, where proteins are selectively retained based on net charge, producing WPI with > 90% protein^40,46,153^. WPH is produced by enzymatic hydrolysis, which breaks proteins into smaller peptides and amino acids^154,155^. Enzymes derived from microbial, plant, or animal sources such as pepsin, trypsin, chymotrypsin, bromelain, alcalase, ficin, fermentation-derived enzymes from bifidobacteria or lactic acid bacteria, are widely utilized in hydrolysis^154^. As proteolytic enzymes functions at specific pH and temperature conditions, they impart distinct nutritional and functional properties, allowing WPH characteristics to be tailored for various applications^154,155^. The protein content of WPH is usually comparable to WPI and varies depending on enzyme used, processing method, and degree of hydrolysis^83,156^. Extensive evidence shows that WP and their peptides support muscle development, as well as cardiovascular, digestive, immune, and nervous system health^154,157,158^. Although bioactive peptides are naturally released from dietary proteins during digestion, their levels are often insufficient to achieve therapeutic effects, necessitating industrial-scale processing^154^. Each protein derivative offers distinct advantages depending on the application^156^. WPC is the most cost-effective derivative for general nutrition and food processing applications due to minimal processing. WPH is particularly suitable as supplements in sports nutrition and for individuals with gastrointestinal sensitivities due to its high digestibility, bioavailability, and reduced allergenicity. WPI is ideal for those with lactose intolerance as it is low in lactose and fat content^156,159^.

## Major bioactive components of whey

### Whey proteins

#### β-lactoglobulin

β-lactoglobulin (β-LG) is the major protein in milk whey, representing 50–55% (3–4 g/L)^41^ of WP and ~10% of total milk proteins^160^. It is a small globular protein with 162 amino acids ( ~ 18.4 kDa), secreted by mammary epithelial cells^41^. Many non-ruminant species, including humans, lack this protein, however, are detected in breast milk of mothers who consume bovine milk^161^. β-LG displays multiple genetic variants, among which A and B are more prevalent and contains two disulfide bonds and one free sulfhydryl group^160^. β-LG exhibits high nutritional value, with an essential amino acid profile comparable that of egg white^162^. Although its full biological function remains underexplored, existing evidence indicates diverse physiochemical attributes linked to its structure, including carrier, functional, and immunomodulatory properties^41,157,163^. Its quaternary structure switches between monomeric, dimeric, or octameric forms depending on surrounding pH^163^. Notably, at pH of 5, β-LG binds and transports calcium and diverse small hydrophobic ligands (retinol, fatty acids, protoporphyrin IX, triacylglycerols, alkanes, aliphatic ketones, aromatic compounds, vitamin D, cholesterol, palmitic acid) which are released during digestion^163^. Its stability at low pH protects these hydrophobic ligands through the stomach, enhancing their bioavailability^163^. Unlike caseins, WPs, including β-LG, are more resistant to intestinal digestion due to its compact globular structure that shields potential epitopes from digestive enzymes. This contributes to reduced bioavailability and allergenicity, especially in infants and some individuals^161^. Industrial processing reduces allergenicity and improves digestibility by altering protein structure, exposing cleavage sites, and releasing bioactive peptides with functional attributes not limited to antioxidant, antimicrobial, anticancer, antihypertensive, and angiotensin-converting enzyme-inhibitory properties^162–165^. Beyond nutrient transport, β-LG aids transfer of passive immunity to newborns, regulates mammary gland phosphorus metabolism, and enhances glutathione synthesis via its cysteine-rich profile, supporting antioxidant defense and muscle growth^40,163^. The multifunctional nature of β-LG supports its use in dietary supplements, nutraceuticals, and as a gelling and stabilizing agent in food processing^41^.

#### Lactalbumin

α-lactalbumin (α-LA) is the second most abundant protein in bovine milk, accounting for 3.5% of total proteins^166^ and 20–25% of WP ( ~ 1.5 g/L)^41^. Human milk contains higher proportions of α-LA compared to bovine milk, accounts for 22–35% of total protein and 36–41% of WP^166,167^. α-LA is synthesized as a pre-protein containing 142 amino acids, of which the first 19 residues form a signal peptide that is cleaved to produce a mature two-domain protein of 123 residues with a molecular weight of 14.2 kDa^41,168^. The two domains (α and β) are separated by a calcium-binding site and linked by two disulfide bridges, while the overall structure is stabilized by four disulfide bonds^169^. α-LA exists as three genetic variants, A, B, and C, where B is the prominent form^112^. It is also synthesized in mammary glands and recognized as a metalloprotein due to its Ca^2+^ binding ability. Other divalent cations (Mg^2+^, Mn^2+^, Na^2+^, Zn^2+^, K^2+^) also compete for the Ca^2+^ binding site, contributing to its stability and versatile functionality^170^. Ca^2+^ enhances its stability against heat, denaturants, and proteases, while Zn^2+^ contrast this effect^167^. Its multiple molecular forms such as the whole protein, peptides of partially hydrolysed protein, and amino acids from fully digested protein, exhibit diverse bioactivities not limited to antioxidant, antimicrobial, antihypertensive, anticancer, immunomodulation, and anti-appetizing effects^165–167,171^. Additionally, α-LA serves as a regulatory subunit of lactose synthase in mammary secretory cells, essential for milk production and infant nutrition^167^. It also transports hydrophobic and bioactive compounds such as fatty acids, retinol, palmitic acid, minerals, vitamins (A, D3, E), as well as polyphenols like curcumin and resveratrol^169,172,173^. The type of compound carried by α-LA is determined by the metal ion it binds^167^. Nutritionally, α-LA is rich in essential amino acids, particularly tryptophan, cysteine, lysine, and branched-chain amino acids (BCAA). Notably, BCAA such as leucine support muscle protein synthesis, while tryptophan and cysteine serve as precursors for serotonin and glutathione, which are essential for antioxidant defense and cognitive functions^118^.

#### Lactoferrin

Lactoferrin (LF) is a non-heme, iron-chelating monomeric glycoprotein, secreted by mucosal epithelial cells into various endocrine secretions. It is particularly abundant in mammary gland secretions, including colostrum, transitional and mature milk^174^. Human milk contains 1–7 g/L of LF, considerably higher than bovine milk (0.03–1.5 g/L)^175,176^. LF accounts for 10-30% of total proteins in human milk whey. Its concentration in colostrum ranges between 5 and 6 g/L, which declines with increasing breastfeeding time to about 1 g/L^177^. LF contains approximately 690–700 amino acids with a molecular weight of 77–91 kDa, depending on its source, glycosylation, lactation stage, feed, age, and breed^174,178^. Its molecular structure comprises a polypeptide chain, folded into two symmetrical lobes (N-lobe and C-lobe) that are 33–41% homologous and connected by α-helix^178^. The N-lobe (residues 1–333) and C-lobe (residues 345–692) each contain two subdomains capable of binding metal ions. LF preferentially binds Fe²⁺/Fe³⁺, but also Zn²⁺, Cu²⁺, and Mn²⁺^175,178^. This strong iron-binding ability enabled LF to exert diverse bioactivities, including intestinal iron absorption, infant intestinal development, wound healing, bone regeneration, antimicrobial, antioxidative, anticarcinogenic, and anti-inflammatory properties^163,176,179,180^. Its antimicrobial potency is extensively assessed, and during the COVID-19 pandemic, LF was proposed as a treatment drug against severe acute respiratory syndrome coronavirus 2 (SARS-CoV)^179^. LF exhibits broad-spectrum antimicrobial activity against various microorganisms, including bacteria (gram-negative, gram-positive, and drug-resistant bacteria), fungi, yeast, virus, and parasites^177^. Its antimicrobial effects are attributed to its two ferric ion-binding lobes. The N-lobe exerts bacteriostatic effects by chelating iron, thereby depriving microorganisms of this essential nutrient. LF also disrupts gram-negative bacteria by binding to membrane components such as LPS and porins^163,179^. LF further inhibits bacterial metabolism and biofilm formation, and its positively charged amino acids can cause cell lysis by binding to anionic microbial surfaces^179^. The C-lobe regulates immune system by controlling iron efficacy through its storage and release under varying pH conditions^181^. Overall, LF modulates both innate and adaptive immune responses to maintain immune homeostasis through the regulating of inflammatory mediators such as interleukins and reactive oxygen species (ROS), as well as the maturation and differentiation of immune cells^177,179^. Due to its nutritional value and broad therapeutic potential, LF has gained significant research interest. While milk is the main source of LF, its low abundance is driving the development of more efficient purification methods and heterologous microbial production^177^.

#### Immunoglobulins

Igs are another major group of glycoproteins in milk, colostrum, and whey. They exist as monomers or polymers with identical light (23–25 kDa) and heavy (50-70 kDa) polypeptide chains linked by disulphide bonds^163,182^. Igs are categorized into five classes (IgG, IgA, IgM, IgE, IgD) based on their structure and molecular function. IgGs ( ~ 160 kDa) exists as monomers, while IgA ( ~ 370 kDa) and IgM (~1000 kDa) as polymers^87^. Igs are primarily secreted from blood serum into milk, colostrum, and other physiological fluids. They are potent immunomodulators with specialized antigen-binding sites that recognize microbial epitopes and promote their clearance via phagocytosis^183^. Bovine milk and colostrum Igs show broad-spectrum antimicrobial activity against pathogens including *Escherichia coli*, *Helicobacter pylori*, *Clostridium difficile*, *Shigella, Cryptosporidium*, Streptococci, Rotavirus, Respiratory syncytial virus, and Human herpes virus^87^. Igs concentrations are over 100-fold higher in bovine colostrum than in mature milk, potentially to provide passive immunity to newborn calves^184^. Notably, IgG (IgG1, IgG2) is the predominant Igs in bovine milk, accounting for 70–80% of total proteins in colostrum, 1-2% in mature milk, and 10–15% in whey^41,87,182^. Conversely, human milk is rich in IgA, comprising ~88.11% of total Igs in colostrum and ~81.65% in mature milk^185^. Available data indicate that Ig profiles vary considerably across studies. For example, bovine colostrum was reported to contain 38.10–67.80 g/L IgG, 0.5–4.4 g/L IgA, 1.1–21 g/L IgM, whereas mature milk contains only 0.257 g/L IgG, 0.04-0.06 g/L IgA, 0.03-0.6 g/L IgM^186^. Other studies report bovine colostrum levels of 15-180 g/L IgG1, 1-3 g/L IgG2, 5 g/L IgM, and 3.5 g/L IgA, compared with 0.35 g/L IgG1, 0.02-0.12 g/L IgG2, 0.04-1 g/L IgM, and 0.05-0.14 g/L IgA in bovine milk^87^. Further, bovine whey contains 0.26–0.50 g/L IgG based on analysis of whey from 100 pooled farm milk samples^187^. Similarly, a recent study reported that total Igs constitute 15.81–16.32% of bovine whey^188^. Filtration techniques are increasingly employed to concentrate Igs from whey, enabling the production of Ig-rich products valuable as functional and nutritional dairy foods^163,189^.

#### Bovine serum albumin

Bovine serum albumin (BSA), a well-characterized serum albumin, is extensively used in biotechnological research for its binding capacity, carrier function, stability, and other key biochemical properties^85^. In general, BSA is a large, globular water-soluble protein with a molecular weight of 66.2–69 kDa. BSA structure is similar to human serum albumin (HSA) and contains three homologous domains, 582 amino acid residues, 17 intermolecular disulphide bridges, and a thiol group at residue 34, which contributes to its structural stability and binding capacity^41,163^. Unlike β-LG and α-LA, BSA is not synthesized in mammary gland but in liver, after which it is released into blood and transferred to milk through passive leakage^163^. BSA is the most abundant protein in FBS (37.1%) and fourth abundant protein in bovine whey (5–10%; 0.3–0.6 g/L)^40,41^. It accounts for 1.2–1.5% of total proteins in milk^41^. As a major constituent of FBS, BSA is often included in SFM to compensate for the absence of serum-derived supportive factors. Although its precise role in SFM remains poorly characterized, as discussed in this review, BSA facilitates the transport of lipids, hormones, and other essential micronutrients to cells^85^. In addition, it contributes to metabolic homeostasis, redox balance via its antioxidant amino acid residues, and osmotic regulation, collectively creating a physiologically favourable microenvironment in cell culture systems^85^. These integrated functions likely underpin its widespread use in SFM, despite the limited mechanistic understanding of its specific contributions to such defined conditions. Accordingly, recent studies have increasingly utilized BSA (0.075 g/L)^52^ and HSA (0.8–5 g/L) (recombinant)^26^ in SFM formulations for cultivated meat research. Media supplemented with these albumins, particularly when combined with other proteins, GFs or enzyme cofactors such as Insulin-Transferrin-Selenium mixture, FGF-2, hepatocyte growth factor, or fetuin, was shown to support cell growth at efficiencies comparable to those achieved with FBS^26,52^. However, the high cost of albumins and GFs, along with the variable efficacy of these formulations, remains a significant challenge for industrial-scale implementation^26,52^.

## Whey enzymes

### Lactoperoxidase

Milk and whey contain various enzymes, including lactoperoxidase (LPO), hydrolases, transferases, lyases, proteases, and lipases, which aid in breakdown of food and catalysis of cellular reactions. LPO is the most abundant glycoprotein enzyme in whey, accounting for 0.25–0.5% of total proteins^41,190^. It was the first enzyme identified in milk and is the second most abundant enzyme in bovine milk (0.03 g/L)^40,191,192^. Significant levels of LPO are also present in colostrum and other mammalian secretions as saliva and tears. LPO consists of 15 cysteines, a heme group, and about 10% (w/w) carbohydrate moieties^166,192^. LPO belongs to the family of peroxidaseenzymes, integral to innate immune system that provides first line of defense against invading pathogens. Specifically, LPO catalyzes the oxidation of certain physiological molecules like thiocyanate ions (SCN⁻), in the presence of hydrogen peroxide (H₂O₂) to produce hypothiocyanite (OSCN^-^). OSCN^-^ then interacts with sulfhydryl (-SH) groups in microbial proteins, disrupting glycolysis and glucose transport to exert antimicrobial action. The LPO (LPO/H_2_O_2_/SCN^-^) system exhibits strong bacteriostatic, bactericidal, antifungal, and antiviral activity against a broad spectrum of microorganisms^165,191,192^. It also protects lactating mammary glands and the intestinal tract of newborn infants from pathogens^191^. Human LPO is a single polypeptide chain with 632 amino acids ( ~ 80 kDa), while bovine LPO contains 612 amino acids ( ~ 78 kDa). Although structurally and functionally similar, bovine LPO exhibits 20-fold higher peroxidase activity^166,190^. Due to its strong antimicrobial properties and broad availability, LPO is widely utilized in food preservation and therapeutic applications^166,191,193^.

## Bioactive peptides and branched-chain amino acids

### Major whey peptide - Glycomacropeptide

Caseinomacropeptide (CMP) constitutes approximately 10–15% (1.2–1.5 g/L) of WP^40,41^. It is a bioactive peptide with 64 amino acids and a molecular weight of ~8.6 kDa^40,41^. CMP is generated during cheesemaking through rennet-mediated cleavage of κ-casein. Rennet hydrolyses κ-casein at the Phe105-Met106 bond, producing para-κ-casein (residues 1-105), which remains in the curd, and glycomacropeptide (GMP; residues 106–169), which is released into whey. κ-casein generates 11 genetic variants, with A and B being predominant in bovine milk. Approximately half of the CMP are glycosylated and are collectively referred to as GMP^188^. It has attracted attention for its immunomodulation, anti-cancer, anti-microbial, prebiotic, antithrombotic, and anti-hypertensive properties^188,194,195^. Previous cellular, animal, and human studies have demonstrated its ability to mitigate intestinal inflammation by regulating innate immune signaling pathways, including Toll-like receptor 4 and Nuclear Factor Kappa B^195^. GMP supports the growth of probiotic bacteria such as bifidobacteria and lactobacilli, while inhibiting pathogens and enterotoxins, including *E. coli, Salmonella typhimurium, Streptococcus mutans, Porphyromonas gingivalis*, Cholera toxin, LPS, and rotavirus^188,195–197^. Its antimicrobial properties are partly attributed to sialic acid residues that act as decoy ligands by binding pathogens that would otherwise interact with host cell sialic acids^195^. Owing to its low phenylalanine content and multifunctional bioactivity, GMP is increasingly explored as a dietary intervention for phenylketonuria, obesity, and inflammatory bowel diseases^198,199^.

### Other minor bioactive peptides derivatives and essential amino acids

Among the 9 essential amino acids in whey, leucine, isoleucine, and valine are branched-chain amino acids (BCAA), which accounts for 18% of total amino acids^200^. BCAAs plays a central role in skeletal muscle protein synthesis, energy metabolism, amino acid uptake, insulin secretion, and maintenance of overall cellular health^200^. In addition to BCAAs, whey serves as an important source of bioactive peptides, which are released during gastrointestinal digestion^157^. Methods such as chemical hydrolysis, microbial fermentation, enzymatic treatment, membrane filtration, ultrasonic, and thermal treatments were widely employed for whey peptide extraction. Notably, enzymatic hydrolysis or microbial fermentation of α-LA and β-LG releases peptides with diverse bioactive properties not limited to antioxidant, antimicrobial, antihypertensive, antidiabetic, and immunomodulatory activities, contributing to gut health, cardiovascular function, and overall immune defense^158,171^. LF-derived peptides exhibits potent antimicrobial activity, encompassing antibacterial, antifungal, antiviral, and antiprotozoal effects, in addition to immunomodulatory, antithrombic, and anticancer properties^158,201^. Moreover, the digestibility and bioavailability of amino acids in whey exceed those of other protein sources, as reflected by its high Protein Digestibility Corrected Amino Acid Score (PDCAAS) and Digestible Indispensable Amino Acid Score (DIAAS), highlighting its superior nutritional quality^154,202^.

## Other minor vital whey components

### Growth factors

GFs and hormones are vital for muscle cell growth, proliferation, and differentiation, functions that are traditionally met through FBS supplementation. Similar to FBS, bovine milk, colostrum, and whey contain several GFs and hormones, particularly IGFs (IGF-I, IGF-II, IGF-binding protein 2), FGFs (FGF1, FGF2), and TGFs (TGF-α, TGF-β, TGF-β1, TGF-β2), which are critical for myogenesis as previously discussed in this review^32,203^. Moreover, milk was reported to contain over 50 distinct GFs and hormones, although at relatively low concentrations ( < 0.001 g/L) compared with Igs (0.8 g/L) and LF (0.02–0.2 g/L)^203^. Like other milk constituents, GFs and hormone concentrations vary depending on lactation stage and detection method. Their levels are highest in colostrum, which later declines over time. The molecular weight of milk GFs ranges between 6.4 and 30 kDa and has neutral to alkaline isoelectric points (pI)^32^. Notably, bovine colostrum was estimated to contain EGF (4 × 10⁻⁶–3.24 × 10⁻⁴ g/L), BTC (2.3 × 10⁻⁶ g/L), IGF-I (3.2 × 10⁻⁵–2 × 10⁻³ g/L), IGF-II (1.5 × 10⁻⁴–6 × 10⁻⁴ g/L), TGF-β (1.01 × 10⁻⁴ g/L), TGF-β1 (1.24 × 10⁻⁵–4.26 × 10⁻⁵ g/L), and TGF-β2 (1.5 × 10⁻⁴–1.15 × 10⁻³ g/L). In contrast, milk contains much lower levels of EGF (2 × 10⁻⁶–1.55 × 10⁻⁴ g/L), Betacellulin (BTC; 1.9 × 10⁻⁶ g/L), IGF-I (2 × 10⁻–1.5 × 10⁻⁴ g/L), IGF-II (2 × 10⁻⁶–1.07 × 10⁻⁴ g/L), TGF-β (4.3 × 10⁻⁶ g/L), TGF-β1 (8 × 10⁻⁷–3.7 × 10⁻⁶ g/L), TGF-β2 (1.37 × 10⁻⁵ g/L), and FGF2 (5 × 10⁻⁷–1 × 10⁻⁶ g/L). Since the mid-1990s, bovine whey has been recognized as an important source of GFs for various applications, including FBS alternative in cell cultures, wound repair, and promotion of gut and bone health^204^. However, isolating of low-abundant and low-molecular mass GFs from complex fluids (colostrum, milk, or whey) remains challenging due to their association high-molecular mass casein and WPs^204,205^. Two decades ago, Francis and colleagues established cation-exchange chromatography-based strategies to efficiently concentrate and elute GFs-rich fractions from bovine cheese whey for use as FBS alternatives in cell cultures^120,204,205^. This method separates GFs with basic pI from major proteins (α-LA, β-LG, BSA), which are acidic^204,205^. However, the eluate may contain LF and LPO due to their basic nature^204^. Accordingly, two GF fractions enriched in LPO or LF were obtained, with the latter had higher mitogenic activity on L6 myoblasts, Balb/c 3T3 cells, and human skin fibroblasts at protein concentrations 2-20-fold lower than required with FBS^205^. Based on these results, 5% serum can be replaced with 1.3, 0.3, and 0.1 g/L of GFs-rich whey fractions in culture media for L6 myoblasts, 3T3 cells, and fibroblasts cultivation, respectively. Similarly, 2.5, 0.5, and 0.2 g/L were sufficient to replace 10% serum^120^. Besides, this study also highlighted that ultrafiltration of acidified whey with 3 to 100 kDa membranes were unable to retain the mitogenic activity of whey filtrate in myoblasts^120^. However, Hossner and Hemm successfully separated colostrum IGF-I (pH 8.0) and IGF-II (pH 5.0 and 8.0) from 30 kDa permeate using ultrafiltration/diafiltration technique with regenerated cellulose and polyether sulphone filters^206^. Furthermore, Maubois and colleagues successfully concentrated TGF-β from native whey using microfiltration technique^207^. The WPI was precipitated by acid treatment (pH 4.0 and 5.5) at varying temperature (25–70 °C), followed by microfiltration using 0.1 μm membrane. The retentate was rich in α-LA and contained 10–50% of the initial protein and 40–100% of the initial TGF-β2 content. Notably, the purity of the fraction increased with raising temperature^207^.

### Vitamins and minerals

Whey contains 12 vitamins, comprising both water-soluble B-complex vitamins (B1, B2, B3, B5, B6, B7, B9, B12), vitamin C, and choline, as well as small quantities of fat-soluble vitamins (A, E)^208^; however, their concentration and composition depends on the whey processing method. An earlier report indicated that the mean vitamin content of sweet and acid whey includes vitamin A, 136 and 107 IU; vitamin C, 0.0141 and 0.0033 g/L; vitamin C, B6 0.0059 and 0.0062 g/L; vitamin B12, 2.4 × 10⁻⁵ and 2.5 × 10⁻⁵ g/L, vitamin E, 0.00063 and 0.00071 g/L; vitamin B1, 0.0051 and 0.0049 g/L; vitamin B2, 0.0214 and 0.0185 g/L; vitamin B5, 0.115 and 0.114 g/L; vitamin B7, 4.3 × 10⁻⁴ and 3.5 × 10⁻⁴ g/L; vitamin B3, 0.0130 and 0.0116 g/L; vitamin B9 1.16 × 10⁻⁴ and 3.32 × 10⁻⁴ g/L; and choline 1.04 and 1.01 g/L^208^. In general, whey serves as a good source of B-group vitamins due to its high-water solubility and association with WP during cheese manufacture, which also imparts its characteristic yellow-green colour^209^. A recent study further reported that between 50 and 91% of B vitamins (B1/B2/B12) and 8–32% of fat-soluble vitamins (A/E) are transferred from milk to whey during processing^209^. Further, commercial whey contains at least 18 minerals as given in Table 1^210,211^. Among these, K (4.689 g/L) and Ca (3.811 g/L) are present in high concentrations, whereas Co (7 × 10⁻⁵ g/L) and V (4 × 10⁻⁵ g/L) are in low amounts^211^.

### Whey supplementation in muscle cell cultivation and myogenic regulation

The effects of whole whey and WP on muscle cell growth, proliferation, and differentiation were previously evaluated across multiple muscle cell models, with a particular focus on cultivated meat applications (Table 2). Primary adult muscle cells and myosatellite cells isolated from bovine or porcine skeletal muscle, as well as immortalized cell line such as mouse C2C12 myoblasts, are widely utilized as cell models for cultivated meat research due to their high proliferative capacity under serum-rich conditions and their ability to readily differentiate into multinucleated myotubes upon serum withdrawal or reduced conditions^9,11,21,51,56^. These cells express key morphological and molecular markers of myogenesis, including genes and proteins of myogenic regulatory factors (MRFs). MRFs comprises a family of four muscle cell-specific helix-loop-helix transcription factors such as MyoD, Myf5, Myogenin (MyoG), and Myogenic regulatory factors 4 (Mrf4)^1^. They play a crucial role in development and functioning of skeletal muscles by precisely controlling every stage of myogenesis, including cell proliferation, cell cycle exit, activation of muscle-specific genes, and subsequent cell-cell fusion for myotube formation through a hierarchical induction of Myf5 and MyoD, followed by MyoG and MRF4^1^. Earlier studies have demonstrated that supplementation of a range of milk and its derivatives such as milk extracts^35^, whey fractions^212^, whey serum^213^, whey peptides^34^, recombinant albumin^26^, individual WP (β-LG, α-LA, BSA, LF) and their mixtures^21,52,214^ have consistently exerted positive regulatory effects on skeletal muscle myogenesis. These compounds were tested across a broad concentration range (0.001–5 g/L or 1-25%)^21,212,214^ and for varying culture durations (1 h to 30 days)^21,26,212,213^ (Table 2). In nearly all studies, the primary effect was enhanced cell proliferation, indicated by increased cell and cell nuclei counts, dsDNA content, cell doubling time, cell membrane integrity, and mitochondrial dehydrogenase activity^21,26,35,52,212^. Treatments including whey fractions, WP mixtures, LF, and milk extracts also promoted myogenic differentiation, as observed from elevated expression of differentiation markers such as Myf5, MyoD, MyoG, myosin heavy chain (MyHC/Myh; Myh1, Myh2, Myh4), citrate synthase (CS), and CK, as well as increased cell-cell fusion, myotube diameter, and hypertrophy^21,212,214^. Myogenesis is typically accompanied by increase in mitochondrial oxidative phosphorylation (OXPHEN) for energy production, which, in turn, is indicative of mitochondrial biogenesis. Notably, CS and CK are key regulators of OXPHEN in differentiating muscle cells and serve as markers of mitochondrial, myocyte, and muscle fiber content^215,216^. Furthermore, colostrum whey fraction-containing culture medium (2.5–5 g/L) supported the cryopreservation of C2C12 cells and their 3D spheroid formation over a 14-days culture period^212^. Spheroids in this medium increased in size and DNA content over time, with significantly larger spheroids observed on day-14, regardless of initial cell seeding density (5000–20,000 cells/well). Conversely, spheroids cultured in FCS-containing medium exhibited only modest size increase between days 7 and 14^212^.

### Edible scaffolds based on whey for cultivated meat production

**Fig. 3:**
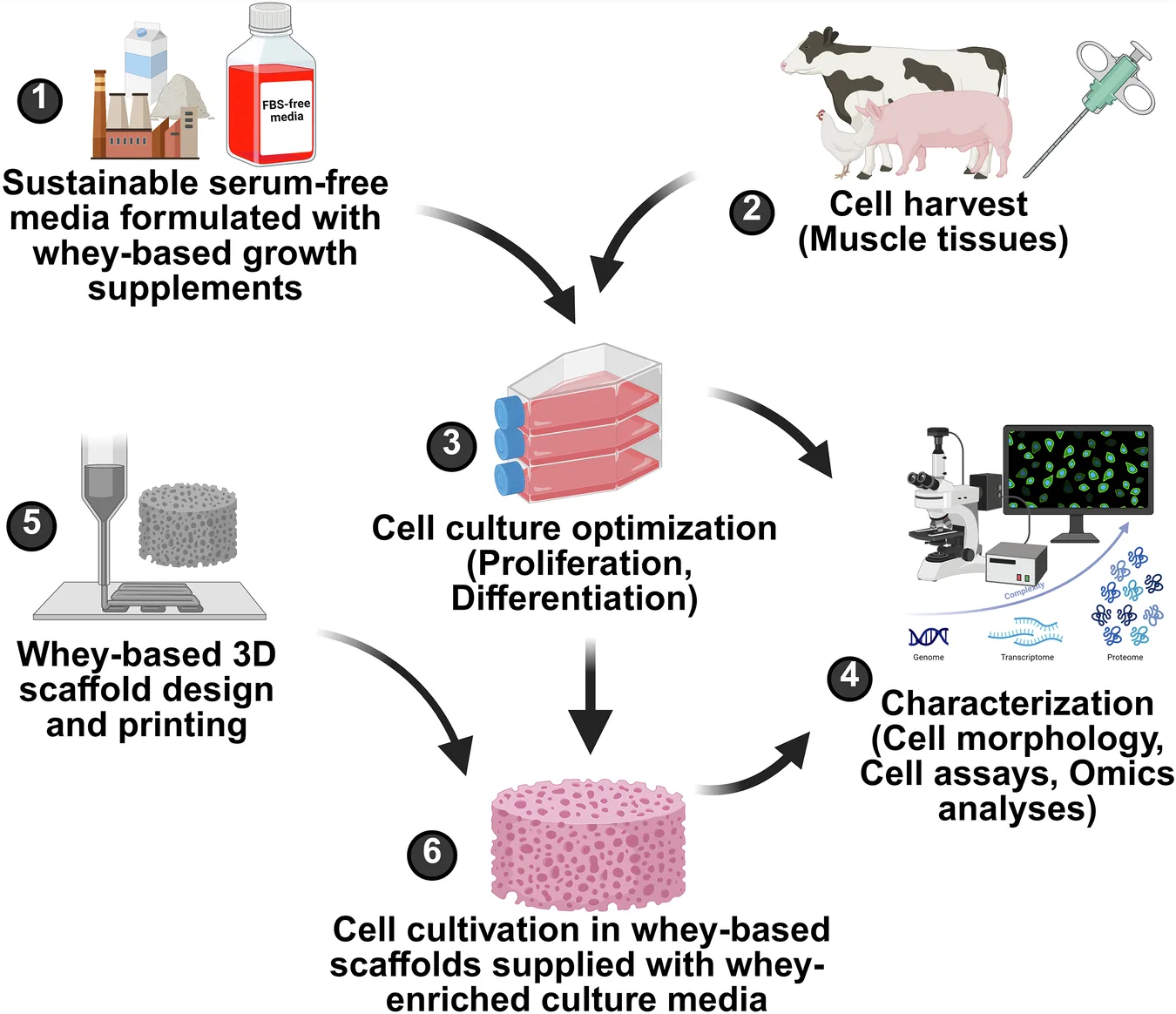
Schematic representation of sustainable cultivated meat production through cell cultivation in whey-based scaffolds with whey-enriched culture media. The process begins with the procurement, processing, and formulation of serum-free whey-enriched culture media (1), followed by the collection of animal cells via biopsy and optimization of culture conditions in whey media (2-3). Edible whey-based 3D scaffolds are then designed, and cells are cultivated within these scaffolds to mimic conventional meat structure, followed by characterization using state-of-the-art cell-based assays and omics technologies (4-6). Figure created using BioRender.com.

The utilization of WP as biocompatible and non-toxic scaffolding materials for cultivated meat production has recently garnered significant scientific interest. Although its application in tissue engineering is not new, its inherent physical-mechanical properties, particularly tensile strength, elasticity, and biocompatibility render them equally advantageous for use as scaffolding materials in cultivated meat systems^48,217,218^. WPI-based hydrogels and composites were formulated with different crosslinkers such as transglutaminase and CaCl₂, either alone or in combination with alginate or glycerol (Table 3), typically at concentrations of 10–20% WPI or β-LG^48,217,218^. In these studies, WP-containing scaffolds supported cell adhesion and proliferation, as evidenced by increased cell density, nuclei count, mitochondrial activity, and improved cell morphology^48,217,218^. Furthermore, myogenic differentiation was enhanced as demonstrated by elevated expression of f-actin, MyHC, and total cytoskeletal protein content over a culture period of 1 to 9 days^217^. Besides their biological performance, these protein-based scaffolds exhibited favourable mechanical and structural properties, including high stiffness, stable swelling ratios, homogenous crosslinking, hydration capacity, dense gel networks, and improved Young’s modulus and zeta potential^48,217,218^. The incorporation of alginate, transglutaminase, glycerol, or CaCl₂ further enhanced gel integrity and resistance to hydrolytic degradation. Compared with other edible scaffolds, such as those made of collagen or gelatin, WP-based scaffolds offer enhanced sustainability alongside advantages in cost-effectiveness, availability, nutritional value, and bioactive properties. However, they may also present challenges, including potential allergenicity and limited tunability of mechanical characteristics^218,219^. As discussed earlier in this section, blending WP with polysaccharides such as alginate can improve both structural integrity and functional performance of the scaffolds^48,218,220^. An overview of the major steps involved in the development of WP-based cell culture media and scaffolds for sustainable cultivated meat production is presented in Fig. 3.

### Conclusion, limitations, and future perspectives

Milk whey is a nutrient-rich biofluid whose biochemical composition and functional properties closely resemble those of FBS, supporting its potential as an alternative supplement for mammalian cell cultures in cultivated meat production. Previous studies indicate that whey proteins are functionally analogous to serum proteins, acting as cell signaling molecules, mediators of enzymatic activities, immunomodulators, carriers of nutrients and bioactive compounds, and contributors to osmotic balance in cell culture systems. Importantly, whey contains families of growth factors and hormones central for muscle myogenesis, including insulin-like growth factors, fibroblast growth factors, and transforming growth factors, which regulate the expression of myogenic regulatory factors. Reflecting these properties, whey derivatives were shown to effectively support muscle cell proliferation, differentiation, long-term maintenance, and cryopreservation. Incorporation of whey into 3D bioprinting strategies has further yielded non-toxic, edible scaffolds with favourable physical-mechanical properties and high biological compatibility. Beyond its nutritional value, whey exhibits functional attributes, not limited to antioxidant, antimicrobial, anticancer, and anti-inflammatory effects, that can be transferred to cultivated meat products via culture media supplementation. Also, meat and dairy products are important sources of vitamin B12, which is essential for neurological function, and deficiency of this vitamin is common among older adults. Standard basal media lacks vitamin B12, but it is naturally present in whey and can be introduced into cultivated meat products. Whey therefore represents a sustainable, ethical, and cost-effective alternative to FBS for serum-free cell culture. Despite these advantages, several limitations remain. Whey and other serum alternatives, including plant-, algal-, or microbial-derived supplements lack significant amounts of extracellular matrix (ECM) proteins such as collagen, laminin, fibrinogen, and fibronectin, which are important for cell-cell and cell-matrix attachment during in vitro cultivation. Hence, during the initial phase of cell seeding, culture media are often supplemented with FBS, or cells are seeded onto culture vessels pre-coated with animal-derived ECM components. Once adequate cellular adhesion is established, FBS-containing media can be replaced with serum-free formulations to support subsequent cell growth, proliferation, and differentiation. In parallel, recombinant and precision fermentation technologies are being explored for the production of animal-free ECM proteins, which could be combined with whey or other serum alternatives to further facilitate the development of fully serum-free cell culture systems. Although whey is relatively cost-effective at the laboratory-scale, its application in industrial-scale cultivated meat production may pose economic challenges due to the operational costs associated with multistage processing required for the purification and enrichment of specific cell-growth promoting fractions from whey. This consideration is particularly important given that culture media is one of the major contributors to overall production costs. In addition, whey is a biologically variable, animal-derived component, and differences in animal origin, lactation stage, diet, and processing conditions may result in batch-to-batch compositional variability, potentially affecting culture reproducibility and process consistency. These challenges highlight the need for standardized sourcing, controlled processing, and routine compositional and functional quality assessments. Additionally, the formulation of whey-based culture media using established techniques, such as selection of growth-promoting whey protein fractions via molecular weight cut-off filtration technique and dose-response evaluation of individual components, enables the development of optimized media. This approach supports standardized culture conditions that promote consistent cell proliferation and differentiation while minimizing batch-to-batch variations, particularly when scaling-up. Furthermore, whey contains milk-derived allergenic proteins, which may raise safety, labelling, and regulatory considerations if residual components persist in the final product, necessitating appropriate allergen risk assessments strategies. Moreover, to date, only a limited number of studies have directly investigated the effects of whey or whey derivatives in muscle cell culture, with most evidence based on the C2C12 cell line. Studies involving agriculturally relevant cells, such as bovine or porcine muscle stem cells are lacking. As C2C12 cells have lower nutritional demands and are easier to culture than primary livestock-derived cells, the ability of whey to effectively substitute FBS in livestock cell culture remains to be validated. In particular, the applicability of whey or other serum alternatives needs systematic comparisons across species, cell types, and differentiation stages. Also, the molecular composition of FBS remains poorly defined, with much of the available data either limited in scope or outdated, which complicates functional comparisons with whey. Future research therefore, should prioritize comprehensive multi-omics characterization, including proteomics, metabolomics, and lipidomics of both FBS and milk whey fractions to enable rigorous, data-driven comparisons. Mechanistic studies are also needed to elucidate how specific whey components influence muscle cell proliferation, differentiation, and scaffold integration. Such analyses are essential for the rational design and optimization of whey-based, serum-free cell culture systems. Overall, the molecular and functional parallel between milk whey and blood serum, combined with its bioactivity and sustainability advantages, underscores its potential as an alternative cell growth supplement for muscle cell cultivation in both food and biomedical applications.

## Data Availability

No datasets were generated or analysed during the current study.

## References

[1] Hernández-Hernández, J. M., García-González, E. G., Brun, C. E. & Rudnicki, M. A. The myogenic regulatory factors, determinants of muscle development, cell identity and regeneration. *Semin. Cell. Dev. Biol.* **72**, 10–18 (2017). 29127045 10.1016/j.semcdb.2017.11.010 PMC5723221

[2] Yashwanth, B. S. et al. In vitro protein expression profile of cultivated muscle cells from Labeo rohita. *Sci. Rep.* **15**, 5859 (2025). 39966484 10.1038/s41598-025-88900-w PMC11836462

[3] Melzener, L. et al. Optimisation of cell fate determination for cultivated muscle differentiation. *Commun. Biol.* **7**, 1493 (2024). 39532984 10.1038/s42003-024-07201-6 PMC11557827

[4] Listrat, A. et al. How muscle structure and composition influence meat and flesh quality. *Sci. World J.* **2016**, 3182746 (2016). 10.1155/2016/3182746 PMC4789028 27022618

[5] Post, M. J. & Hocquette, J. F. in New Aspects of Meat Quality: From Genes to Ethics. (ed. P.P. P.) 425-441 (Woodhead Publishing, Duxford, United Kingdom and Cambridge, MA; 2017).

[6] World Food and Agriculture – Statistical Yearbook. (FAO, Rome, Italy; 2023).

[7] Hocquette, Chriki, J. F., Fournier, S. & Ellies-Oury, D. M.P. Review: Will “cultured meat” transform our food system towards more sustainability? *Animal* **19**, 101145 (2025). 38670917 10.1016/j.animal.2024.101145

[8] McNamara, E. & Bomkamp, C. Cultivated meat as a tool for fighting antimicrobial resistance. *Nat. Food* **3**, 791–794 (2022). 37117880 10.1038/s43016-022-00602-y

[9] Jang, M., Scheffold, J., Rost, L. M., Cheon, H. & Bruheim, P. Serum-free cultures of C2C12 cells show different muscle phenotypes which can be estimated by metabolic profiling. *Sci. Rep.* **12**, 827 (2022). 35039582 10.1038/s41598-022-04804-z PMC8764040

[10] Florini, J. R., Ewton, D. Z. & Magri, K. A. Hormones, growth factors, and myogenic differentiation. *Annu. Rev. Physiol.* **53**, 201–216 (1991). 2042960 10.1146/annurev.ph.53.030191.001221

[11] Yaffe, D. & Saxel, O. A myogenic cell line with altered serum requirements for differentiation. *Differentiation* **7**, 159–166 (1977). 558123 10.1111/j.1432-0436.1977.tb01507.x

[12] Brindley, D. A. et al. Peak serum: implications of serum supply for cell therapy manufacturing. *Regen. Med.* **7**, 7–13 (2012). 22168489 10.2217/rme.11.112

[13] van der Valk, J. et al. Fetal Bovine Serum (FBS): Past - Present - Future. *ALTEX* **35**, 99–118 (2018). 28800376 10.14573/altex.1705101

[14] Chelladurai, K. S. et al. Alternative to FBS in animal cell culture - An overview and future perspective. *Heliyon* **7**, e07686 (2021). 34401573 10.1016/j.heliyon.2021.e07686 PMC8349753

[15] Logarušić, M. et al. Protein hydrolysates from flaxseed oil cake as a media supplement in CHO cell culture. *Resources* **10**, 59 (2021).

[16] Giron-Calle, J. et al. Chickpea protein hydrolysate as a substitute for serum in cell culture. *Cytotechnology* **57**, 263–272 (2008). 19003183 10.1007/s10616-008-9170-z PMC2570005

[17] Amirvaresi, A., Sarkarat, R., Jones, C., Shahsavari, A. & Ovissipour, R. Sustainable alternatives to fetal bovine serum: evaluating the role of plant and insect protein isolates in serum-free media for bovine satellite cell proliferation in cultivated meat production. *ACS Food Sci. Technol.* **5**, 1614–1624 (2025).

[18] Yamanaka, K., Haraguchi, Y., Takahashi, H., Kawashima, I. & Shimizu, T. Development of serum-free and grain-derived-nutrient-free medium using microalga-derived nutrients and mammalian cell-secreted growth factors for sustainable cultured meat production. *Sci. Rep.* **13**, 498 (2023). 36627406 10.1038/s41598-023-27629-w PMC9832167

[19] Ng, J. Y. et al. Chlorella vulgaris extract as a serum replacement that enhances mammalian cell growth and protein expression. *Front. Bioeng. Biotechnol.* **8**, 564667 (2020). 33042965 10.3389/fbioe.2020.564667 PMC7522799

[20] Venkatesan, M. et al. Recombinant production of growth factors for application in cell culture. *iScience* **25**, 105054 (2022). 36157583 10.1016/j.isci.2022.105054 PMC9489951

[21] Sundaram, T. S. et al. Milk whey as a sustainable alternative growth supplement to fetal bovine serum in muscle cell culture. *J. Dairy Sci.* **108**, 4749–4760 (2025). 39986454 10.3168/jds.2024-25449

[22] Andreassen, R. C., Pedersen, M. E., Kristoffersen, K. A. & Ronning, S. B. Screening of by-products from the food industry as growth promoting agents in serum-free media for skeletal muscle cell culture. *Food Funct.* **11**, 2477–2488 (2020). 32134068 10.1039/c9fo02690h

[23] Cheli, F. et al. in Cell-Based Meat in the European Union and Beyond. (eds. G. Formici, M. C. Mancini & L. Scaffardi) 19-51 (Springer, Cham, 2026).

[24] Malomo, S. A. & Aluko, R. E. A comparative study of the structural and functional properties of isolated hemp seed (Cannabis sativa L.) albumin and globulin fractions. *Food Hydrocoll.* **43**, 743–752 (2015).

[25] Stout, A. J. et al. A Beefy-R culture medium: Replacing albumin with rapeseed protein isolates. *Biomaterials* **296**, 122092 (2023). 36965281 10.1016/j.biomaterials.2023.122092 PMC10111969

[26] Stout, A. J. et al. Simple and effective serum-free medium for sustained expansion of bovine satellite cells for cell cultured meat. *Commun. Biol.* **5**, 466 (2022). 35654948 10.1038/s42003-022-03423-8 PMC9163123

[27] Khan, I. et al. Innovations, challenges, and regulatory pathways in cultured meat for a sustainable future. *Foods* **14**, 2 (2025). 10.3390/foods14183183 PMC12469421 41008156

[28] Zao, A., Nogueira, W. V., Rondan, F. S. & Scaglioni, P. T. Cultured meat: A multidimensional review of technological, nutritional, ethical, and regulatory advances (2020-2025). *J. Food Sci.* **91**, e70915 (2026). 41684290 10.1111/1750-3841.70915 PMC12902808

[29] Besediuk, V., Yatskov, M., Korchyk, N., Kucherova, A. & Maletskyi, Z. Whey - From waste to a valuable resource. *J. Agric. Food Res.* **18**, 101280 (2024).

[30] Sun, Y., Wang, C., Sun, X. & Guo, M. Proteomic analysis of differentially expressed whey proteins in Guanzhong goat milk and Holstein cow milk by iTRAQ coupled with liquid chromatography-tandem mass spectrometry. *J. Dairy Sci.* **103**, 8732–8740 (2020). 32713692 10.3168/jds.2020-18564

[31] Yang, M. et al. Comparative proteomic exploration of whey proteins in human and bovine colostrum and mature milk using iTRAQ-coupled LC-MS/MS. *Int. J. Food Sci. Nutr.* **68**, 671–681 (2017). 28276902 10.1080/09637486.2017.1279129

[32] Gauthier, S. F., Pouliot, Y. & Maubois, J. Growth factors from bovine milk and colostrum: composition, extraction and biological activities. *Le. Lait.* **86**, 99–125 (2006).

[33] Xu, R., Xiao, Y., Zhang, Y. & Zhao, X. The role of whey protein in myogenic differentiation. *Ital. j. food cci.* **34**, 43–49 (2022).

[34] Knowles, S., Gilmartin, S., Arranz, E., O’Brien, N. & Giblin, L. Effect of bioavailable whey peptides on C2C12 muscle cells. *Proceedings* **11**, 35 (2019).

[35] Shima, A., Oda, H. & Takeuchi, S. Milk-derived extract for skeletal muscle cell cultivation. *Food Biosci.* **66**, 106218 (2025).

[36] Wall, S. K., Gross, J. J., Kessler, E. C., Villez, K. & Bruckmaier, R. M. Blood-derived proteins in milk at start of lactation: Indicators of active or passive transfer. *J. Dairy Sci.* **98**, 7748–7756 (2015). 26298756 10.3168/jds.2015-9440

[37] R, T. et al. Milk and serum proteomes in subclinical and clinical mastitis in Simmental cows. *J Proteomics* **244**, 104277 (2021). 10.1016/j.jprot.2021.104277 34044168

[38] Alonso-Fauste, I. et al. Proteomic characterization by 2-DE in bovine serum and whey from healthy and mastitis affected farm animals. *J. Proteom.* **75**, 3015–3030 (2012). 10.1016/j.jprot.2011.11.035 22193514

[39] Wellnitz, O. & Bruckmaier, R. M. Invited review: The role of the blood-milk barrier and its manipulation for the efficacy of the mammary immune response and milk production. *J. Dairy Sci.* **104**, 6376–6388 (2021). 33773785 10.3168/jds.2020-20029

[40] Minj, S. & Anand, S. Whey proteins and its derivatives: bioactivity, functionality, and current applications. *Dairy* **1**, 233–258 (2020).

[41] Mehra, R. et al. Whey proteins processing and emergent derivatives: An insight perspective from constituents, bioactivities, functionalities to therapeutic applications. *J. Funct. Food* **87**, 104760 (2021).

[42] Caffarelli, C. et al. Cow’s milk allergy in breastfed infants: what we need to know about mechanisms, management, and maternal role. *Nutrients* **17**, 1787 (2025). 40507056 10.3390/nu17111787 PMC12156988

[43] Eurostat, European Comission. How much milk does the EU produce? https://ec.europa.eu/eurostat/web/products-eurostat-news/w/edn-20240531-1. (2022).

[44] Buchanan, D., Martindale, W., Romeih, E. & Hebishy, E. Recent advances in whey processing and valorisation: Technological and environmental perspectives. *Int. J. Dairy Technol.* **76**, 291–312 (2023).

[45] Smithers, G. W. Whey-ing up the options – Yesterday, today and tomorrow. *Int. Dairy J.* **48**, 2–14 (2015).

[46] Aguero, R., Bringas, E., Román, M. F. S., Ortiz, I. & Ibañez, R. Membrane processes for whey proteins separation and purification. *A Rev. Curr. Org. Chem.* **21**, 1740–1752 (2017).

[47] Woo, H. D., Moon, T. W., Gunasekaran, S. & Ko, S. Determining the gelation temperature of beta-lactoglobulin using in situ microscopic imaging. *J. Dairy Sci.* **96**, 5565–5574 (2013). 23871379 10.3168/jds.2013-6786

[48] Tahir, I., Foley, C. & Floreani, R. Whey protein isolate and β-lactoglobulin-modified alginate hydrogel scaffolds enhance cell proliferation for cultivated meat applications. *Foods* **14**, 2534 (2025). 40724354 10.3390/foods14142534 PMC12294790

[49] Montano, M. in Translational Biology in Medicine 103-128 (Woodhead Publishing, 2014).

[50] Gu, H. et al. Scaling cultured meat: challenges and solutions for affordable mass production. *Compr. Rev. Food Sci. Food Saf.* **24**, e70221 (2025). 40635127 10.1111/1541-4337.70221 PMC12241508

[51] Katayama, T., Chigi, Y. & Okamura, D. The ensured proliferative capacity of myoblast in serum-reduced conditions with Methyl-beta-cyclodextrin. *Front. Cell Dev. Biol.* **11**, 1193634 (2023). 37250904 10.3389/fcell.2023.1193634 PMC10213241

[52] Skrivergaard, S. et al. A simple and robust serum-free media for the proliferation of muscle cells. *Food Res. Int.* **172**, 113194 (2023). 37689947 10.1016/j.foodres.2023.113194

[53] Wang, Y., Zhuang, D., Munawar, N., Zan, L. & Zhu, J. A rich-nutritious cultured meat via bovine myocytes and adipocytes co-culture: Novel Prospect for cultured meat production techniques. *Food Chem.* **460**, 140696 (2024). 39111042 10.1016/j.foodchem.2024.140696

[54] Seo, K., Suzuki, T., Kobayashi, K. & Nishimura, T. Adipocytes suppress differentiation of muscle cells in a co-culture system. *Anim. Sci. J.* **90**, 423–434 (2019). 30585366 10.1111/asj.13145

[55] Yin, W., Sun, Z., McClements, D. J., Jin, Z. & Qiu, C. Advances in 3D scaffold materials and fabrication techniques for cultured meat production: A review. *Food Biosci.* **73**, 107564 (2025).

[56] Yaffe, D. Retention of differentiation potentialities during prolonged cultivation of myogenic cells. *Proc. Natl. Acad. Sci. Usa.* **61**, 477–483 (1968). 5245982 10.1073/pnas.61.2.477 PMC225183

[57] Eagle, H. Nutrition needs of mammalian cells in tissue culture. *Science* **122**, 501–514 (1955). 13255879 10.1126/science.122.3168.501

[58] Eagle, H. Amino acid metabolism in mammalian cell cultures. *Science* **130**, 432–437 (1959). 13675766 10.1126/science.130.3373.432

[59] Eagle, H. The specific amino acid requirements of a mammalian cell (strain L) in tissue culture. *J. Biol. Chem.* **214**, 839–852 (1955). 14381421

[60] Eagle, H. The minimum vitamin requirements of the L and HeLa cells in tissue culture, the production of specific vitamin deficiencies, and their cure. *J. Exp. Med.* **102**, 595–600 (1955). 13271674 10.1084/jem.102.5.595 PMC2136536

[61] van der Valk, J. Fetal bovine serum-a cell culture dilemma. *Science* **375**, 143–144 (2022). 35025663 10.1126/science.abm1317

[62] Kierans, S. J. & Taylor, C. T. Glycolysis: A multifaceted metabolic pathway and signaling hub. *J. Biol. Chem.* **300**, 107906 (2024). 39442619 10.1016/j.jbc.2024.107906 PMC11605472

[63] Furuichi, Y. et al. Excess glucose impedes the proliferation of skeletal muscle satellite cells under adherent culture conditions. *Front. Cell Dev. Biol.* **9**, 640399 (2021). 33732705 10.3389/fcell.2021.640399 PMC7957019

[64] Luo, W., Ai, L., Wang, B. F. & Zhou, Y. High glucose inhibits myogenesis and induces insulin resistance by down-regulating AKT signaling. *Biomed. Pharmacother.* **120**, 109498 (2019). 31634780 10.1016/j.biopha.2019.109498

[65] Newsholme, P., Procopio, J., Lima, M. M., Pithon-Curi, T. C. & Curi, R. Glutamine and glutamate-their central role in cell metabolism and function. *Cell Biochem. Funct.* **21**, 1–9 (2003). 12579515 10.1002/cbf.1003

[66] Cruzat, V., Rogero, M. M., Keane, K. N., Curi, R. & Newsholme, P. Glutamine: metabolism and immune function, supplementation and clinical translation. *Nutrients* **10**, 1564 (2018). 30360490 10.3390/nu10111564 PMC6266414

[67] Wilmore, D. W. Glutamine. (National Academies Press, Washington, D.C. (US); (1999).

[68] Zielke, H. R., Ozand, P. T., Tildon, J. T., Sevdalian, D. A. & Cornblath, M. Growth of human diploid fibroblasts in the absence of glucose utilization. *Proc. Natl. Acad. Sci. USA* **73**, 4110–4114 (1976). 1069299 10.1073/pnas.73.11.4110 PMC431346

[69] Jomova, K. et al. Essential metals in health and disease. *Chem. Biol. Interact*. **367**, 6 (2022). 10.1016/j.cbi.2022.110173 36152810

[70] Prabhu, A. & Gadgil, M. Trace metals in cellular metabolism and their impact on recombinant protein production. *Process Biochem* **110**, 251–262 (2021).

[71] Yang, Z. & Xiong, H. in Biomedical Tissue Culture. (eds. L. Ceccherini-Nelli & B. Matteoli) 3-18 (IntechOpen, London; 2012).

[72] Smith, G. D., Swain, J. E. & Pool, T. B. Embryo Culture: Methods and Protocols, Vol. 912. (Humana Press, 2012).

[73] Schnellbaecher, A., Binder, D., Bellmaine, S. & Zimmer, A. Vitamins in cell culture media: Stability and stabilization strategies. *Biotechnol. Bioeng.* **116**, 1537–1555 (2019). 30793282 10.1002/bit.26942 PMC6594077

[74] Lin, J. C. & Gant, N. in Magnetic Resonance Spectroscopy. (eds. C. Stagg & D. Rothman) 104-110 (Academic Press, 2014).

[75] Fischer, A. Amino-acid metabolism of tissue cells in vitro. *Biochem. J.* **43**, 491–497 (1948). 16748439 10.1042/bj0430491 PMC1274763

[76] Puck, T. T., Cieciura, S. J. & Robinson, A. Genetics of somatic mammalian cells. III. Long-term cultivation of euploid cells from human and animal subjects. *J. Exp. Med.* **108**, 945–956 (1958). 13598821 10.1084/jem.108.6.945 PMC2136918

[77] Gstraunthaler, G. Alternatives to the use of fetal bovine serum: Serum-free cell culture. *Altern. Anim. Exp.* **20**, 275–281 (2003). 14671707

[78] Weber, T. et al. Fetal bovine serum: how to leave it behind in the pursuit of more reliable science. *Front. Toxicol.* **7**, 1612903 (2025). 40861932 10.3389/ftox.2025.1612903 PMC12371577

[79] Florini, J. R. & Roberts, S. B. A serum-free medium for the growth of muscle cells in culture. *Vitro* **15**, 983–992 (1979). 10.1007/BF02619157 94034

[80] Gstraunthaler, G., Lindl, T. & van der Valk, J. A plea to reduce or replace fetal bovine serum in cell culture media. *Cytotechnology* **65**, 791–793 (2013). 23975256 10.1007/s10616-013-9633-8 PMC3967615

[81] Lee, D. Y. et al. Review of the current research on fetal bovine serum and the development of cultured meat. *Food Sci. Anim. Resour.* **42**, 775–799 (2022). 36133630 10.5851/kosfa.2022.e46 PMC9478980

[82] Hemeda, H., Giebel, B. & Wagner, W. Evaluation of human platelet lysate versus fetal bovine serum for culture of mesenchymal stromal cells. *Cytotherapy* **16**, 170–180 (2014). 24438898 10.1016/j.jcyt.2013.11.004

[83] Zheng, X. et al. Proteomic analysis for the assessment of different lots of fetal bovine serum as a raw material for cell culture. Part IV. Application of proteomics to the manufacture of biological drugs. *Biotechnol. Prog.* **22**, 1294–1300 (2006). 17022666 10.1021/bp060121o

[84] Schalich, K. M., Herren, A. W. & Selvaraj, V. Analysis of differential strategies to enhance detection of low-abundance proteins in the bovine serum proteome. *Anim. Sci. J.* **91**, e13388 (2020). 32578273 10.1111/asj.13388

[85] Francis, G. L. Albumin and mammalian cell culture: implications for biotechnology applications. *Cytotechnology* **62**, 1–16 (2010). 20373019 10.1007/s10616-010-9263-3 PMC2860567

[86] Washington, I. M. & Hoosier, G. V. in The Laboratory Rabbit, Guinea Pig, Hamster, and Other Rodents. (eds. M. A. Suckow, K. A. Stevens & R. P. Wilson) 57–116 (Academic Press, 2011).

[87] Feeney, S., Morrin, S. T., Joshi, L. & Hickey, R. M. in Novel Proteins for Food, Pharmaceuticals and Agriculture: Sources, Applications and Advances. (ed. M. Hayes) 291−314 (John Wiley & Sons, USA; 2018).

[88] Santibanez, J. F. & Kocic, J. Transforming growth factor-beta superfamily, implications in development and differentiation of stem cells. *Biomol. Concepts* **3**, 429–445 (2012). 25436548 10.1515/bmc-2012-0015

[89] Dayton, W. R. & Hathaway, M. R. Myogenic cell proliferation and differentiation. *Poult. Sci.* **70**, 1815–1822 (1991). 1924098 10.3382/ps.0701815

[90] Velloso, C. P. Regulation of muscle mass by growth hormone and IGF-I. *Br. J. Pharmacol.* **154**, 557–568 (2008). 18500379 10.1038/bjp.2008.153 PMC2439518

[91] Frantz, C., Stewart, K. M. & Weaver, V. M. The extracellular matrix at a glance. *J. Cell Sci.* **123**, 4195–4200 (2010). 21123617 10.1242/jcs.023820 PMC2995612

[92] Hynes, R. O. Integrins: bidirectional, allosteric signaling machines. *Cell Press* **110**, 673–687 (2002). 10.1016/s0092-8674(02)00971-6 12297042

[93] Costa, L. R. R. & Chapman, A. in Manual of Clinical Procedures in the Horse. (eds. L. R. R. Costa & M. R. Paradis) 59-66 (John Wiley and Sons, Inc., Hoboken, NJ; 2017).

[94] Gonzales, G. L. in Annual Convention of the AAEP, Vol. 47 (AAEP PROCEEDINGS, 2001).

[95] Vilanova, X. M., Beaver, B., Uldahl, M. & Turner, P. V. Recommendations for ensuring good welfare of horses used for industrial blood, serum, or urine production. *Anim. (Basel)* **11**, 1466 (2021). 10.3390/ani11051466 PMC8161321 34065236

[96] Freshney, R. I. Culture of Animal Cells: A Manual of Basic Technique and Specialized Applications. (John Wiley & Sons, Inc., Hoboken, NJ; 2011).

[97] Levy, E. M. The ability of horse serum to support an in vitro antibody response. *J. Immunol. Methods* **36**, 181–183 (1980). 7000910 10.1016/0022-1759(80)90042-3

[98] Allen, R. E., Dodson, M. V. & Luiten, L. S. Regulation of skeletal muscle satellite cell proliferation by bovine pituitary fibroblast growth factor. *Exp. Cell Res.* **152**, 154–160 (1984). 6714317 10.1016/0014-4827(84)90239-8

[99] Franke, J., Abs, V., Zizzadoro, C. & Abraham, G. Comparative study of the effects of fetal bovine serum versus horse serum on growth and differentiation of primary equine bronchial fibroblasts. *BMC Vet. Res.* **10**, 119 (2014). 24886635 10.1186/1746-6148-10-119 PMC4040117

[100] Minonzio, G. M. & Linetsky, E. The use of fetal bovine serum in cellular products for clinical applications: commentary. *CellR4* **2**, e1307 (2014).

[101] Nielsen, O. B. & Hawkes, P. W. Fetal bovine serum and the slaughter of pregnant cows: animal welfare and ethics. *BioProcess. J*. **18**, 11–13 (2019).

[102] Zitterer, I. & Paulsen, P. Slaughter of pregnant cattle at an Austrian abattoir: prevalence and gestational age. *Anim. (Basel)* **11**, 2474 (2021). 10.3390/ani11082474 PMC8388674 34438931

[103] Versteegen, R. J., Murray, J. & Doelger, S. Animal welfare and ethics in the collection of fetal blood for the production of fetal bovine serum. *ALTEX* **38**, 319–323 (2021). 33871036 10.14573/altex.2101271

[104] Malikides, N., Hodgson, J. L., Rose, R. J. & Hodgson, D. R. Cardiovascular, haematological and biochemical responses after large volume blood collection in horses. *Vet. J.* **162**, 44–55 (2001). 11409929 10.1053/tvjl.2001.0583

[105] van der Valk, J. & Gstraunthaler, G. Fetal Bovine Serum (FBS) - A pain in the dish? *Altern. Lab. Anim.* **45**, 329–332 (2017). 29313704 10.1177/026119291704500611

[106] Pecora, A. et al. Analysis of irradiated Argentinean fetal bovine serum for adventitious agents. *J. Vet. Diagn. Invest.* **32**, 892–897 (2020). 32814516 10.1177/1040638720951556 PMC7649553

[107] Paim, W. P. et al. Virome characterization in commercial bovine serum batches-A potentially needed testing strategy for biological products. *Viruses* **13**, 2425 (2021). 34960693 10.3390/v13122425 PMC8705701

[108] Xia, H., Vijayaraghavan, B., Belak, S. & Liu, L. Detection and identification of the atypical bovine pestiviruses in commercial foetal bovine serum batches. *PLoS One* **6**, e28553 (2011). 22174836 10.1371/journal.pone.0028553 PMC3234271

[109] Baker, M. Reproducibility: Respect your cells! *Nature* **537**, 433–435 (2016). 27629646 10.1038/537433a

[110] Pecora, A., Aguirreburualde, M. S. P., Ridpath, J. F. & Santos, M. J. D. Molecular characterization of pestiviruses in fetal bovine sera originating from Argentina: evidence of circulation of HoBi-like viruses. *Front. Vet. Sci.* **6**, 359 (2019). 31681812 10.3389/fvets.2019.00359 PMC6805694

[111] Haley, N. J. & Richt, J. A. Classical bovine spongiform encephalopathy and chronic wasting disease: two sides of the prion coin. *Anim. Dis.* **3**, 24 (2023).

[112] Ena, J. Prions: who should worry about them? *Arch. Med. Res.* **36**, 622–627 (2005). 16216643 10.1016/j.arcmed.2005.02.004

[113] Varshney, M., Waggoner, P. S., Montagna, R. A. & Craighead, H. G. Prion protein detection in serum using micromechanical resonator arrays. *Talanta* **80**, 593–599 (2009). 19836525 10.1016/j.talanta.2009.07.032

[114] Hawkes, P. W. Fetal bovine serum: geographic origin and regulatory relevance of viral contamination. *Bioresour. Bioprocess.* **2**, 34 (2015).

[115] Camphora, A. L. & Pucca, M. B. Horses used for large-scale production of immunoglobulins: an inter-species approach. *Int. Anim. Health J.* **8**, 12–16 (2022).

[116] Pucca, M. B. & Camphora, A. L. The potential risks of equine serum therapy in transmitting new infectious diseases: lessons from a post-pandemic era. *Front. Public Health* **12**, 1366929 (2024). 38420034 10.3389/fpubh.2024.1366929 PMC10899399

[117] Paim, W. P. et al. Characterization of the viral genomes present in commercial batches of horse serum obtained by high-throughput sequencing. *Biologicals* **61**, 1–7 (2019). 31447377 10.1016/j.biologicals.2019.08.005

[118] Fang, C. Y., Wu, C. C., Fang, C. L., Chen, W. Y. & Chen, C. L. Long-term growth comparison studies of FBS and FBS alternatives in six head and neck cell lines. *PLoS One* **12**, e0178960 (2017). 28591207 10.1371/journal.pone.0178960 PMC5462426

[119] Lee, H. S., Yoon, H. J. & Lee, S. O. Fetal bovine serum substitution efficacy of mealworm (Tenebrio molitor) protein hydrolysates and its physicochemical properties. *Food Res. Int.* **208**, 116204 (2025). 40263843 10.1016/j.foodres.2025.116204

[120] Smithers, G. W. et al. New opportunities from the isolation and utilization of whey proteins. *J. Dairy Sci.* **79**, 1454–1459 (1996). 8880470 10.3168/jds.s0022-0302(96)76504-9

[121] Jayme, D. W., Epstein, D. A. & Conrad, D. R. Fetal bovine serum alternatives. *Nature* **334**, 547–548 (1988). 3405300 10.1038/334547a0

[122] Liu, S., Yang, W., Li, Y. & Sun, C. Fetal bovine serum, an important factor affecting the reproducibility of cell experiments. *Sci. Rep.* **13**, 1942 (2023). 36732616 10.1038/s41598-023-29060-7 PMC9894865

[123] Guidance Document on Good In Vitro Method Practices (GIVIMP), OECD Series on Testing and Assessment, No. 286. 10.1787/9789264304796-en. *OECD Publishing*, *Paris* (2018).

[124] Meth, J. L. & Schoenfeld, A. R. Higher percentage of horse serum in culture media blocks attachment of PC12 cells. *Biotechniques* **67**, 256–258 (2019). 31621377 10.2144/btn-2019-0073 PMC7031818

[125] Ditz, T. et al. Phospholipase A2 products predict the hematopoietic support capacity of horse serum. *Differentiation* **105**, 27–32 (2019). 30554008 10.1016/j.diff.2018.12.002

[126] European Medicines Agency, European Union. Guideline on the use of bovine serum in the manufacture of human biological medicinal products (CPMP/BWP/1793/02). https://www.ema.europa.eu/en/use-bovine-serum-manufacture-human-biological-medicinal-products-scientific-guideline. (2013).

[127] Requirements for ingredients of animal origin used for production of biologics. United States Code of Federal Regulations (9 CFR 113.53). https://www.ecfr.gov/current/title-9/chapter-I/subchapter-E/part-113/subject-group-ECFRa4cbfb362190bc0/section-113.53. (2025).

[128] Current Good Manufacturing Practice for Animal Cells, Tissues, and Cell and Tissue-Based Products. U. S. Food and Drug Administration. https://www.fda.gov/regulatory-information/search-fda-guidance-documents/cvm-gfi-253-current-good-manufacturing-practice-animal-cells-tissues-and-cell-and-tissue-based. (2022).

[129] van der Valk, J. et al. Optimization of chemically defined cell culture media-replacing fetal bovine serum in mammalian in vitro methods. *Toxicol. Vitr.* **24**, 1053–1063 (2010). 10.1016/j.tiv.2010.03.016 20362047

[130] Taylor, W. G., Dworkin, R. A., Pumper, R. W. & Evans, V. J. Biological efficacy of several commercially available peptones for mammalian cells in culture. *Exp. Cell Res.* **74**, 275–279 (1972). 4561337 10.1016/0014-4827(72)90505-8

[131] Schenzle, L. et al. Low-cost food-grade alternatives for serum albumins in FBS-free cell culture media. *Sci. Rep.* **15**, 15296 (2025). 40312489 10.1038/s41598-025-99603-7 PMC12045953

[132] Mark Post’s Cultured Beef. *New Harvest* https://www.new-harvest.org/blog/mark-post-cultured-beef (2015).

[133] Lee, D. Y. et al. Analysis of commercial fetal bovine serum (FBS) and its substitutes in the development of cultured meat. *Food Res. Int.* **174**, 113617 (2023). 37986472 10.1016/j.foodres.2023.113617

[134] Fan, G. et al. Animal-derived free hydrolysate in animal cell culture: Current research and application advances. *J. Tissue Eng.* **15**, 20417314241300388 (2024). 39649943 10.1177/20417314241300388 PMC11624555

[135] Jo, Y., Kweon, H., Ji, S. D., Kim, J. G. & Kim, K. Y. Silk Protein as a Fetal Bovine Serum Substitute for Animal Cell Culture. *MBL* **47**, 487–497 (2019).

[136] Liu, L. et al. Systematic evaluation of sericin protein as a substitute for fetal bovine serum in cell culture. *Sci. Rep.* **6**, 31516 (2016). 27531556 10.1038/srep31516 PMC4987615

[137] Mathews, M. G. R. et al. Biochemical and functional characterization of heat-inactivated coelomic fluid from earthworms as a potential alternative for fetal bovine serum in animal cell culture. *Sci. Rep.* **14**, 5606 (2024). 38453984 10.1038/s41598-024-56169-0 PMC10920628

[138] Average prices of liquid whey cooled for industrial use in Milano, Italy. *Monza Brianza Lodi Chamber of Commerce* https://www.clal.it/en/index.php?section=siero_milano (2026).

[139] Marella, C., Muthukumarappan, K. & Metzger, L. E. Evaluation of commercially available, wide-pore ultrafiltration membranes for production of alpha-lactalbumin-enriched whey protein concentrate. *J. Dairy Sci.* **94**, 1165–1175 (2011). 21338782 10.3168/jds.2010-3739

[140] Yver, A. L., Bonnaillie, L. M., Yee, W., McAloon, A. & Tomasula, P. M. Fractionation of whey protein isolate with supercritical carbon dioxide-process modeling and cost estimation. *Int. J. Mol. Sci.* **13**, 240–259 (2012). 22312250 10.3390/ijms13010240 PMC3269684

[141] Zandona, E., Blazic, M. & Jambrak, A. R. Whey utilization: Sustainable uses and environmental approach. *Food Technol. Biotechnol.* **59**, 147–161 (2021). 34316276 10.17113/ftb.59.02.21.6968 PMC8284110

[142] Lappa, I. K. et al. Cheese whey processing: integrated biorefinery concepts and emerging food applications. *Foods* **8**, 347 (2019). 31443236 10.3390/foods8080347 PMC6723228

[143] Smithers, G. W. Whey and whey proteins—From ‘gutter-to-gold. *Int. Dairy J.* **18**, 695–704 (2008).

[144] Paladii, I. V., Vrabie, E. G., Sprinchan, K. G. & Bologa, M. K. Whey: Review. Part 2. Treatment processes and methods. *Surf. Engin. Appl. Electrochem.* **57**, 651–666 (2021).

[145] OECD-FAO Agricultural Outlook 2025-2034. (2025).

[146] Goulding, D. A., Fox, P. F. & O’Mahony, J. A. in Milk Proteins: From Expression to Food, Edn. 3. (eds. M. Boland & H. Singh) 21–98 (Academic Press, USA; 2020).

[147] Tsermoula, P., Khakimov, B., Nielsen, J. H. & Engelsen, S. B. WHEY - The waste-stream that became more valuable than the food product. *TIFS* **118**, 230–241 (2021).

[148] Olvera-Rosales, L. B. et al. Bioactive peptides of whey: obtaining, activity, mechanism of action, and further applications. *Crit. Rev. Food Sci. Nutr.* **63**, 10351–10381 (2023). 35612490 10.1080/10408398.2022.2079113

[149] Ryan, M. P. & Walsh, G. The biotechnological potential of whey. *RESB* **15**, 479–498 (2016).

[150] Fernández-Gutiérrez, D. et al. Biovalorization of saccharides derived from industrial wastes such as whey: a review. *Rev. Environ. Sci. Biotechnol.* **16**, 147–174 (2017).

[151] Mirzakulova, A. et al. Whey: composition, processing, application, and prospects in functional and nutritional beverages—A review. *Foods* **14**, 3245 (2025). 41008217 10.3390/foods14183245 PMC12470128

[152] Rocha-Mendoza, D. et al. Invited review: Acid whey trends and health benefits. *J. Dairy Sci.* **104**, 1262–1275 (2021). 33358165 10.3168/jds.2020-19038

[153] Onwulata, C. & Huth, P. Whey Processing, Functionality and Health Benefits. (Wiley, 2009).

[154] Dullius, A., Goettert, M. I. & de Souza, C. F. V. Whey protein hydrolysates as a source of bioactive peptides for functional foods – Biotechnological facilitation of industrial scale-up. *J. Funct. Foods* **42**, 58–74 (2018).

[155] Li, J. & Zhu, F. Whey protein hydrolysates and infant formulas: Effects on physicochemical and biological properties. *Compr. Rev. Food Sci. Food Saf.* **23**, e13337 (2024). 38578124 10.1111/1541-4337.13337

[156] Tang, C., Xi, T., Zheng, J. & Cui, X. Chemical properties of whey protein in protein powders and its impact on muscle growth in athletes: A review. *Nat. Prod. Commun.* **20**, 1–17 (2025).

[157] Korhonen, H. & Pihlanto, A. Bioactive peptides: Production and functionality. *Int. Dairy J.* **16**, 945–960 (2006).

[158] Gauthier, S. F., Pouliot, Y. & Saint-Sauveur, D. Immunomodulatory peptides obtained by the enzymatic hydrolysis of whey proteins. *Int. Dairy J.* **16**, 1315–1323 (2006).

[159] Jeewanthi, R. K. C., Lee, N. & Paik, H. Improved functional characteristics of whey protein hydrolysates in food industry. *Korean J. Food Sci.* **35**, 350–359 (2015). 10.5851/kosfa.2015.35.3.350 PMC4662358 26761849

[160] Le Maux, S., Bouhallab, S., Giblin, L., Brodkorb, A. & Croguennec, T. Bovine beta-lactoglobulin/fatty acid complexes: binding, structural, and biological properties. *Dairy Sci. Technol.* **94**, 409–426 (2014). 25110551 10.1007/s13594-014-0160-y PMC4121524

[161] Picariello, G. et al. Antibody-independent identification of bovine milk-derived peptides in breast-milk. *Food Funct.* **7**, 3402–3409 (2016). 27396729 10.1039/c6fo00731g PMC4981550

[162] Chatterton, D. E. W., Smithers, G., Roupas, P. & Brodkorb, A. Bioactivity of β-lactoglobulin and α-lactalbumin—Technological implications for processing. *Int. Dairy J.* **16**, 1229–1240 (2006).

[163] Madureira, A. R., Pereira, C. I., Gomes, A. M. P., Pintado, M. E. & Malcata, F. X. Bovine whey proteins - Overview on their main biological properties. *Food Res. Int.* **40**, 1197–1211 (2007).

[164] Tavares, T. G. & Malcata, F. X. in Bioactive Food Peptides in Health and Disease. (eds. B. Hernandez-Ledesma & C. Hsieh) 75-114 (IntechOpen, London; 2013).

[165] Korhonen, H. J. in Functional Foods, Edn. 2. (ed. M. Saarela) 471-511 (Woodhead Publishing, 2011).

[166] Morrin, S. T., Buck, R. H., Farrow, M. & Hickey, R. M. Milk-derived anti-infectives and their potential to combat bacterial and viral infection. *J. Funct. Food*. **81**, 25 (2021).

[167] Permyakov, E. A. alpha-Lactalbumin, amazing calcium-binding protein. *Biomolecules* **10**, 1210 (2020). 32825311 10.3390/biom10091210 PMC7565966

[168] Brew, K. in Encyclopedia of Dairy Sciences, Edn. 2. (eds. et al.) 780-800 (Academic Press, USA; 2011).

[169] Mohammadi, F. & Moeeni, M. Study on the interactions of trans-resveratrol and curcumin with bovine alpha-lactalbumin by spectroscopic analysis and molecular docking. *Mater. Sci. Eng. C.* **50**, 358–366 (2015). 10.1016/j.msec.2015.02.007 25746281

[170] Permyakov, E. A. & Berliner, L. J. α-Lactalbumin: structure and function. *FEBS Lett.* **473**, 269–274 (2000). 10818224 10.1016/s0014-5793(00)01546-5

[171] Brandelli, A., Daroit, D. J. & Corrêa, A. P. F. Whey as a source of peptides with remarkable biological activities. *Food Res. Int.* **73**, 149–161 (2015).

[172] Chen, W. et al. Comparison of carrying mechanism between three fat-soluble vitamins and alpha-lactalbumin: Effects on structure and physicochemical properties of alpha-lactalbumin. *Food Hydrocoll.* **116**, 106662 (2021).

[173] Shi, R. et al. Insight into binding behavior, structure, and foam properties of α-lactalbumin/glycyrrhizic acid complex in an acidic environment. *Food Hydrocoll.* **125**, 107411 (2022).

[174] Dyrda-Terniuk, T. & Pomastowski, P. The multifaceted roles of bovine lactoferrin: molecular structure, isolation methods, analytical characteristics, and biological properties. *J. Agric. Food Chem.* **71**, 20500–20531 (2023). 38091520 10.1021/acs.jafc.3c06887 PMC10755757

[175] Hong, R. et al. A review of the biological activities of lactoferrin: mechanisms and potential applications. *Food Funct.* **15**, 8182–8199 (2024). 39027924 10.1039/d4fo02083a

[176] Jiang, R., Du, X. & Lonnerdal, B. Effects of forming lactoferrin-milk protein complexes on lactoferrin functionality and intestinal development in infancy. *Nutrients* **16**, 4077 (2024). 39683471 10.3390/nu16234077 PMC11644007

[177] Cui, S. et al. Recent advances and prospects in purification and heterologous expression of lactoferrin. *Food Bioeng.* **1**, 58–67 (2022).

[178] García-Montoya, I. A., Cendón, T. S., Arévalo-Gallegos, S. & Rascón-Cruz, Q. Lactoferrin a multiple bioactive protein: An overview. *Biochimica et. Biophysica Acta (BBA) - Gen. Subj.* **1820**, 226–236 (2012). 10.1016/j.bbagen.2011.06.018 PMC7127262 21726601

[179] Gruden, Š & Ulrih, N. P. Diverse mechanisms of antimicrobial activities of lactoferrins, lactoferricins, and other lactoferrin-derived peptides. *Int. J. Mol. Sci.* **22**, 11264 (2021). 34681923 10.3390/ijms222011264 PMC8541349

[180] Ashraf, M. F. et al. Nutraceutical and Health-Promoting Potential of Lactoferrin, an Iron-Binding Protein in Human and Animal: Current Knowledge. *Biol. Trace Elem. Res.* **202**, 56–72 (2024). 37059920 10.1007/s12011-023-03658-4 PMC10104436

[181] Ng, Z. J. et al. Lactoferrin in health and disease: A review of its bioavailability and evidence-based benefits across study models. *Trends Food Sci. Technol.* **160**, 22 (2025).

[182] Korhonen, H., Marnila, P. & Gill, H.S. Milk immunoglobulins and complement factors. *Br. J. Nutr.* **84**, S75–S80 (2000). 10.1017/s0007114500002282 11242450

[183] Park, Y. W. & Nam, M. S. Bioactive peptides in milk and dairy products: A review. *Korean J. Food Sci. Anim. Resour.* **35**, 831–840 (2015). 26877644 10.5851/kosfa.2015.35.6.831 PMC4726964

[184] Gomes, R. D. S. et al. Bovine colostrum: A source of bioactive compounds for prevention and treatment of gastrointestinal disorders. *NFS J.* **25**, 1–11 (2021).

[185] Rio-Aige, K. et al. The Breast Milk Immunoglobulinome. *Nutrients* **13**, 23 (2021). 10.3390/nu13061810 PMC8230140 34073540

[186] Arslan, A. et al. Bovine colostrum and its potential for human health and nutrition. *Front. Nutr.* **8**, 651721 (2021). 34235166 10.3389/fnut.2021.651721 PMC8255475

[187] Valk-Weeber, R. L., Eshuis-de Ruiter, T., Dijkhuizen, L. & van Leeuwen, S. S. Quantitative analysis of bovine whey glycoproteins using the overall N-linked whey glycoprofile. *Int. Dairy J.* **110**, 104814 (2020). 10.1021/acs.jafc.0c00959 PMC7304067 32438810

[188] Karimidastjerd, A. & Gulsunoglu-Konuskan, Z. Biological, functional and nutritional properties of caseinomacropeptide from sweet whey. *Crit. Rev. Food Sci. Nutr.* **63**, 4261–4273 (2023). 34802348 10.1080/10408398.2021.2000360

[189] Ostertag, F. & Hinrichs, J. Enrichment of Lactoferrin and Immunoglobulin G from Acid Whey by Cross-Flow Filtration. *Foods* **12**, 23 (2023). 10.3390/foods12112163 PMC10252284 37297408

[190] Gupta, C. & Prakash, D. Therapeutic potential of milk whey. *Beverages* **3**, 31 (2017).

[191] Ozhan, H. K., Duman, H., Bechelany, M. & Karav, S. Lactoperoxidase: properties, functions, and potential applications. *Int. J. Mol. Sci.* **26**, 5055 (2025). 40507866 10.3390/ijms26115055 PMC12154331

[192] Kussendrager, K. D. & van Hooijdonk, A. C. Lactoperoxidase: physico-chemical properties, occurrence, mechanism of action and applications. *Br. J. Nutr.* **84**, S19–S25 (2000). 11242442 10.1017/s0007114500002208

[193] Garcia, H. S., López-Hernandez, A. & Jr., C. G. H. in Comprehensive Biotechnology Vol. 4, Edn. 3. (ed. M. Moo-Young) 608-617 (Elsevier 2017).

[194] Neelima, Sharma, R., Rajput, Y. S. & Mann, B. Chemical and functional properties of glycomacropeptide (GMP) and its role in the detection of cheese whey adulteration in milk: a review. *Dairy Sci. Technol*. **93**, 21–43 (2013). 10.1007/s13594-012-0095-0 PMC3567326 23396893

[195] Adler, S. M., Paluska, M. R., Svoboda, K. R. & Dallas, D. C. Immunomodulatory bioactivities of glycomacropeptide. *J. Funct. Foods* **115**, 106084 (2024).

[196] Malkoski, M. et al. Kappacin, a novel antibacterial peptide from bovine milk. *Antimicrob. Agents Chemother.* **45**, 2309–2315 (2001). 11451690 10.1128/AAC.45.8.2309-2315.2001 PMC90647

[197] Inagaki, M. et al. Bovine kappa-casein inhibits human rotavirus (HRV) infection via direct binding of glycans to HRV. *J. Dairy Sci.* **97**, 2653–2661 (2014). 24612801 10.3168/jds.2013-7792

[198] Tosi, M. et al. Glycomacropeptide-based protein substitutes for children with phenylketonuria in Italy: A nutritional comparison. *Nutrients* **16**, 956 (2024). 38612990 10.3390/nu16070956 PMC11013192

[199] Sawin, E. A. et al. Glycomacropeptide is a prebiotic that reduces Desulfovibrio bacteria, increases cecal short-chain fatty acids, and is anti-inflammatory in mice. *Am. J. Physiol. Gastrointest. Liver Physiol.* **309**, G590–G601 (2015). 26251473 10.1152/ajpgi.00211.2015 PMC4593820

[200] Neinast, M., Murashige, D. & Arany, Z. Branched chain amino acids. *Annu. Rev. Physiol.* **81**, 139–164 (2019). 30485760 10.1146/annurev-physiol-020518-114455 PMC6536377

[201] Saubenova, M. et al. Bioactive peptides derived from whey proteins for health and functional beverages. *Fermentation* **10**, 359 (2024).

[202] Herreman, L., Nommensen, P., Pennings, B. & Laus, M. C. Comprehensive overview of the quality of plant- and animal-sourced proteins based on the digestible indispensable amino acid score. *Food Sci. Nutr.* **8**, 5379–5391 (2020). 33133540 10.1002/fsn3.1809 PMC7590266

[203] Michaelidou, A. & Steijns, J. Nutritional and technological aspects of minor bioactive components in milk and whey: Growth factors, vitamins and nucleotides. *Int. Dairy J.* **16**, 1421–1426 (2006).

[204] Pouliot, Y. & Gauthier, S. F. Milk growth factors as health products: some technological aspects. *Int. Dairy J.* **16**, 1415–1420 (2006).

[205] Francis, G. L., Regester, G. O., Webb, H. A. & Ballard, F. J. Extraction from cheese whey by cation-exchange chromatography of factors that stimulate the growth of mammalian cells. *J. Dairy Sci.* **78**, 1209–1218 (1995). 7673513 10.3168/jds.S0022-0302(95)76740-6

[206] Hossner, K. L. & Yemm, R. S. Improved recovery of insulin-like growth factors (IGFs) from bovine colostrum using alkaline diafiltration. *Biotechnol. Appl. Biochem.* **32**, 161–166 (2000). 11115387 10.1042/ba20000056

[207] Maubois, J., Fauquant, J., Jouan, P. & Bourtourault, M., Vol. US8937043B2. (ed. U. S. P. A. T. OFFICE) (United States; 2003).

[208] Glass, L. & Hedrick, T. I. Nutritional composition of sweet- and acid-type dry wheys. II. vitamin, mineral, and calorie contents. *J. Dairy Sci.* **60**, 190–196 (1977).

[209] Mirzakulova, A. et al. Whey: composition, processing, application, and prospects in functional and nutritional beverages-A review. *Foods* **14**, 3245 (2025). 41008217 10.3390/foods14183245 PMC12470128

[210] Wong, N. P., LaCroix, D. E. & McDonough, F. E. Minerals in whey and whey fractions. *J. Dairy Sci.* **61**, 1700–1703 (1978). 570573 10.3168/jds.S0022-0302(78)83790-4

[211] González-Weller, D. et al. Proteins and minerals in whey protein supplements. *Foods* **12**, 2238 (2023). 37297488 10.3390/foods12112238 PMC10252490

[212] Baldeweg, T. H. et al. Dairy byproducts as sustainable alternatives to FCS in 2D and 3D skeletal muscle cell cultures. *Bioresour. Bioprocess* **12**, 101 (2025). 40982101 10.1186/s40643-025-00938-w PMC12454707

[213] Kontos, N. J. et al. The effects of creatine monohydrate and/or whey protein on the muscle protein synthesis and anabolic signaling responses in non-stressed C2C12 murine myotubes. *Physiologia* **5**, 17 (2025).

[214] Kitakaze, T. et al. Lactoferrin promotes murine C2C12 myoblast proliferation and differentiation and myotube hypertrophy. *Mol. Med. Rep.* **17**, 5912–5920 (2018). 29436684 10.3892/mmr.2018.8603 PMC5866037

[215] Remels, A. H. V. et al. Regulation of mitochondrial biogenesis during myogenesis. *Mol. Cell Endocrinol.* **315**, 113–120 (2010). 19804813 10.1016/j.mce.2009.09.029

[216] Kelley, D. E., He, J., Menshikova, E. V. & Ritov, V. B. Dysfunction of mitochondria in human skeletal muscle in type 2 diabetes. *Diabetes* **51**, 2944–2950 (2002). 12351431 10.2337/diabetes.51.10.2944

[217] Pei, J. et al. Design of edible whey protein isolate hydrogels with cell adhesion via a two-step crosslinking method for cultured meat scaffolds. *Food Hydrocoll.* **168**, 111562 (2025).

[218] Foley, C. & Floreani, R. A. Impact of whey protein on the physico-mechanical properties of alginate dialdehyde scaffolds and cell adhesion for cultivated meat applications. *Food Hydrocoll.* **170**, 111749 (2025). 41311461 10.1016/j.foodhyd.2025.111749 PMC12652571

[219] Cava, E. et al. Investigating the Health Implications of Whey Protein Consumption: A Narrative Review of Risks, Adverse Effects, and Associated Health Issues. *Healthcare (Basel)* **12**, 31 (2024). 10.3390/healthcare12020246 PMC10815430 38255133

[220] Charron, P. N., Tahir, I., Foley, C., White, G. & Floreani, R. A. Whey protein isolate composites as potential scaffolds for cultivated meat. *ACS Appl. Bio Mater.* **7**, 2153–2163 (2024). 38502811 10.1021/acsabm.3c00944

[221] Brunner, D. et al. Serum-free cell culture: the serum-free media interactive online database. *ALTEX* **27**, 53–62 (2010). 20390239 10.14573/altex.2010.1.53

[222] Schroeder, H. W. Jr. & Cavacini, L. Structure and function of immunoglobulins. *J. Allergy Clin. Immunol.* **125**, S41–S52 (2010). 20176268 10.1016/j.jaci.2009.09.046 PMC3670108

[223] Onay-Ucar, E. et al. Comparison of antioxidant capacity, protein profile and carbohydrate content of whey protein fractions. *Food Chem.* **150**, 34–40 (2014). 24360416 10.1016/j.foodchem.2013.10.119

[224] Lee, D. Y. et al. The roles of media ingredients in muscle cell culture for cultured meat production—A mini-review. *Future Foods* **10**, 100403 (2024).

[225] Vaghela, M. & Kilara, A. Lipid composition of whey protein concentrates manufactured commercially and in the laboratory. *J. Dairy Sci.* **79**, 1172–1183 (1996).

[226] Jouan, P.-N., Pouliot, Y., Gauthier, S. F. & Laforest, J.-P. Hormones in bovine milk and milk products: A survey. *Int. Dairy J.* **16**, 1408–1414 (2006).

[227] Comin, A. et al. Technical note: Direct enzyme immunoassay of progesterone in bovine milk whey. *J. Dairy Sci.* **88**, 4239–4242 (2005). 16291615 10.3168/jds.S0022-0302(05)73110-6

[228] Aranda, P., Sanchez, L., Perez, M. D., Ena, J. M. & Calvo, M. Insulin in bovine colostrum and milk: evolution throughout lactation and binding to caseins. *J. Dairy Sci.* **74**, 4320–4325 (1991). 1787200 10.3168/jds.S0022-0302(91)78627-X

[229] Parant, F. et al. Selenium discrepancies in fetal bovine serum: impact on cellular selenoprotein expression. *Int. J. Mol. Sci*. **25**, 7261 (2024). 10.3390/ijms25137261 PMC11242189 39000368

[230] Çelik, K. Whey Every Aspect. (Tudás Alapítvány, 2020).

[231] Banaszek, A. et al. The effects of whey vs. pea protein on physical adaptations following 8-weeks of high-intensity functional training (HIFT): A pilot study. *Sports (Basel)* **7**, 12 (2019). 10.3390/sports7010012 PMC6358922 30621129

[232] Kerasioti, E. et al. Antioxidant effects of whey protein on muscle C2C12 cells. *Food Chem.* **155**, 271–278 (2014). 24594185 10.1016/j.foodchem.2014.01.066

